# Study of Bohr Mottelson Hamiltonian with minimal length effect for Woods-Saxon potential and its thermodynamic properties

**DOI:** 10.1016/j.heliyon.2021.e06861

**Published:** 2021-05-03

**Authors:** A. Suparmi, L.K. Permatahati, S. Faniandari, Y. Iriani, A. Marzuki

**Affiliations:** Physics Department, Universitas Sebelas Maret, Surakarta, Indonesia

**Keywords:** Bohr Mottelson Hamiltonian equation, Minimal length effect, Woods-Saxon potential, Hypergeometric method, Thermodynamic properties

## Abstract

The Bohr Mottelson Hamiltonian with the variable of *β* collective shape for the Woods-Saxon potential in the rigid deformed nucleus for γ=0 and the X(3) model was investigated in the presence of the minimal length formalism. The Bohr Mottelson Hamiltonian was solved approximately by proposing a new wave function. The q-deformed hyperbolic potential concept such that the rigid deformed nucleus of the Bohr Mottelson equation in the minimal length formalism for Woods-Saxon potential was used, so that the equation was reduced to the form of Schrodinger-like equation with cotangent hyperbolic potential. The hypergeometric method was used to obtain the energy spectra equation and the unnormalized wave function of the system. The results showed that the energy spectra were affected by the quantum number, the minimal length parameter, and the atomic mass. The larger mass of the atom affected the energy spectra to decrease, the increase of the values of the minimal length affected the increase of the energy spectra of all atoms. The energy spectra were used to determine the thermodynamic properties including the partition function, mean energy, specific heat, free energy, and entropy of the quantum system with the help of the imaginary error function.

## 1 Introduction

The concepts of critical point symmetries in the nuclear structure are a very interesting theoretical framework. The symmetries of the critical point [1], [2] associated to the transitions of the shape phase have been considered to the analytical solutions of the Bohr Mottelson Hamiltonian [3]. In the quantum system, the Bohr Mottelson Hamiltonian [4] has an important role in the description of the collective behavior of the atomic nuclei [5], [6] and to describe the energy of the nucleus. The solution for Bohr-Mottelson Hamiltonian equation has been used the deformed even-even nuclei with differential potentials models [7]. The corresponding deformed nucleus to the excitation energy has been used to describe vibrational and rotational of the nucleus is named by quadrupole [8]. The nucleus at low excitation energy and it is considered to be in the rotation motion is called a rigid deformed nucleus. For investigating the collective properties of the atomic nuclei, the Bohr Mottelson Hamiltonian is expressed in terms of the two internal variables (β,γ) and three Euler angles (θ,ϕ,φ) [9]. The *β* variable associates to a nucleus deformation radially. The *γ* variable corresponds to symmetric angle [9], [10] and the three Euler angles determine the orientation of the ellipsoid in the space [10], [11]. The symmetric case with γ=0 is called the axially symmetric case [8], [12] which takes place in the rigid deformed nucleus [9], [12] and associates to the prolate deformed nucleus [13], while in the case for γ=π6 associates to the oblate and called as the triaxial symmetric case [14].

Several types of critical point symmetric cases have been studied, namely X(5), E(5), Z(5), and Y(5). The X(5) critical point symmetries describing the first-order phase transition between spherical and prolate deformed nuclei [14], [15], [16], the E(5) type of critical point symmetries representing the second-order phase transition between *γ*-unstable nuclei and spherical [17], [18], [19], the Z(5) type of critical point symmetries corresponding to the transition from prolate to oblate shape [12], and the Y(5) critical point symmetries referring to the shape phase transition. The transition in the Y(5) critical point symmetries is from the axial rotor to the triaxial rotor [20].

Different potential models have been applied in the Bohr Mottelson equations such as infinite square well potential [17], [21], Morse potential [22], [23], Kratzer potential [5], [23], Davidson potential [10], Eckart potential [24], [25], Woods–Saxon [26], Hulthen potential [27], etc. Various methods have been used to solve The Bohr Mottelson Hamiltonian equation with different potentials, such as the Nikiforov-Uvarov method [9], Asymptotic Iteration Method [28], and Supersymmetric quantum mechanics method [24].

In this paper, we use the Woods-Saxon potential introduced by Woods and Saxon to study proton (20 MeV) elastic scattering with a heavy nucleus [7], [29]. It provided big flexibility and apparently in treating the obtained results [30]. This potential was applied to understand the nuclear energy level spacing and properties of electron distributions in atoms, nuclei, and atomic clusters as well [31], [32], [33]. The Woods Saxon potential and some of the infinite well potential have been used in the critical point symmetries related to shape phase transitions [34], [35]. The Woods–Saxon potential has been used in nuclear physics as a single-particle potential extensively [36]. The Woods–Saxon potential is an exponential type of potential and it is the most realistic potentials in nuclear physics which has a short-range. The Woods–Saxon potential is used to determine the energy levels of single-particle and the interactions of the nucleus-nucleus [37], [38], therefore it is stated as one of the most useful models referring to its roles [4], [39]. Besides, the conceptual understanding of the interactions between the nucleus-nucleus for the resonant and bound states is constructed by the exact solutions of the Woods–Saxon potential for the wave equations. These are the reasons to study the Woods-Saxon type potential.

Recently, the problems of the quantum mechanical implying generalized modified commutation relations which include a minimal length have attracted a great deal of attention. The commutation relations are described by the Heisenberg uncertainty principle especially between momentum and position operators. The minimal distance in the scale Planck length was observed [7], [40] by considering the quantum gravity effect on the Heisenberg uncertainty principle. The GUP or called minimal length is the corrected Heisenberg uncertainty principle caused by the quantum gravity [7], [40], [41]. By considering the deformed canonical commutation relation, the minimal length concept can be incorporated in the study of the physical systems [41]. The Bohr Mottelson Hamiltonian equation with minimal length effect has been studied by Chabab et al. [41], Alimohammadi and Hassanabadi [7], Hassanabadi et al. [42], and Suparmi et al. [24] for certain potential models. The potential model used in the Bohr Mottelson equation within the minimal length formalism that has been solved was infinite square well potential either solved approximately using new wave function [41] or using alternative solution [7]. Furthermore, the Bohr Mottelson Hamiltonian equation with minimal length effect for the Davidson [41] and Hulthen potential [43] was solved approximately using (binomial) expansion to the first-degree expansion.

The application of the new wave function in the solution of the Bohr Mottelson equation within minimal length formalism is intended to get rid of the extra term in the form of quadratic Laplacian that arises in the Bohr Mottelson equation due to the effect of minimal length. As a by product, the application of the new wave function in the Bohr Mottelson within minimal length formalism causes the change of the potential term into an unusual form such that the equation could not be solved analytically. To get the analytical solution, the unusual potential has been manipulated mathematically using (binomial) expansion to the first-degree expansion [41], [43].

In the current study, the q deformed hyperbolic potential [44] with its spatial translation [45] concepts will be applied to manipulate the potential change such that The Bohr Mottelson equation with minimal length effect reduces to the Schrodinger like equation. A few potentials that could be applicable such as Yukawa, Hulthen, Woods-Saxon, and modified Eckart potentials. By the use of q deformed hyperbolic potential and its spatial translation concepts in manipulating unusual potential terms will cause The Bohr Mottelson equation to reduce to the usual Schrodinger-like equation that could be solved analytically. In this approximate solution, the value of q, as a deformed hyperbolic potential parameter, has to be positive. On the other hand, the value of q is a function of atomic mass, energy, minimal length parameter, and diffusivity, therefore in this study, we explore the numerical calculation of energy spectra for the various values of radial and orbital quantum numbers, and minimal length parameter that provides the values of q are positives.

In this work, we studied the energy spectra and the wave function of the Bohr Mottelson equation in the rigid deformed nucleus for γ=0 and the X(3) model within the minimal length formalism for the Woods-Saxon potential with the *β* function by using the hypergeometric method. By applying the q deformed hyperbolic concept and translation [44], [45] the approximate solution of Bohr Mottelson equation within minimal length formulation for the Woods-Saxon potential reduced to a Schrodinger-like equation with cotangent hyperbolic potential that was solved by using the hypergeometric method. In this approximation, the value of the q deformed parameter of hyperbolic function potential has to be positive.

Meanwhile, the thermodynamic properties of the potential type were determined from the energy equation and it was expressed in terms of error function or imaginary error function. The partition function equation then was used to determine the thermodynamic properties equations. The thermodynamic properties have been studied by Dong and Cruz Irisson for the modified Rosen Morse potential [46], Ikot et al. for general molecular potentials [47], Oyewumi et al. for shifted Den Fan potential [48], Okorie and Ibekwe for modified Yukawa potential [49], Ikhdair et al. for Poschl Teller potential [50], Song et al. for the sodium dimer [51], and Suparmi et al. for q-deformed modified Posch Teller plus Manning Rosen non-central potential [52].

The structure of this paper is as follows, the approximate solution of Bohr Mottelson Hamiltonian with minimal length effect is presented in section 2, section 3 contains the brief description of the effect of the minimal length on Bohr Mottelson Hamiltonian equation, the review of the hypergeometric method is in section 4. Section 5 present the results and discussion about the energy spectra, the un-normalized wave function, and thermodynamic properties. The last section presents the conclusion.

## 2 The approximate solution of Bohr Mottelson Hamiltonian equation with minimal length effect

In quantum mechanics, the particle dynamics associates to the particle's momentum and position. The commutation relation between momentum and position is expressed using the Heisenberg Uncertainty Principle, defined as [12], [42]

(1) $$\left[\overset{ˆ}{x},\overset{ˆ}{p}\right]\geq ih$$

with $\overset{ˆ}{x}$ is the position operator, $\overset{ˆ}{p}$ is the momentum operator, *i* is the imaginer number, and $\frac{h}{2\pi }=\hbar$ (*h* is the Planck constant). The study of GUP was inspired by noncommutative geometry [53], [54] in the quantum gravity [55], [56] and the string theory context [57], [58]. The GUP or the concept of minimal length was introduced in the study of physical systems by considering the deformed canonical relation [53] given by

(2) $$\left[X,P\right]=i\hbar \left(1+\alpha_{\mathrm{ML}}p^{2}\right)$$

with $\alpha_{\mathrm{ML}}$ is the minimal length parameter which is in the range $0\leq \alpha_{\mathrm{ML}}\leq 1$, *P* is the momentum quantity in high energy. While, *p* is momentum at lower energy. The deformed uncertainty of canonical commutation relation leads to the deformed uncertainty relation expressed as

(3) $$\Delta X\Delta P=\frac{\hbar }{2}\left(1+\alpha_{\mathrm{ML}}{\left(\Delta p\right)}^{2}\right)$$

From equation (3), we have the minimal length formulation

(4) $${\left(\Delta X\right)}_{\mathrm{ML}}=\hbar \sqrt{\alpha_{\mathrm{ML}}}$$

The position and the momentum operators commute each other, that is

(5) $${\overset{ˆ}{X}}_{i}={\overset{ˆ}{x}}_{i}$$

(6) $${\overset{ˆ}{P}}_{i}=\left(1+\alpha_{\mathrm{ML}}{\overset{ˆ}{p}}^{2}\right){\overset{ˆ}{p}}_{i}$$

According to string theory, ${\overset{ˆ}{p}}_{i}$ and ${\overset{ˆ}{P}}_{i}$ shown in equation (6) are momentum operator at low energy and momentum operator at high energy, respectively. The squared of the momentum operator at low energy is formed by [24]

(7) $${\overset{ˆ}{p}}^{2}=-\hbar^{2}\Delta$$

while, by substituting equation (7) into equation (6) will be obtained the squared of the momentum operator at high energy, given as

(8) $${\overset{ˆ}{P}}^{2}=-\hbar^{2}\left(1-2\alpha_{\mathrm{ML}}\hbar^{2}\Delta \right)\Delta$$

where Δ is the Laplacian operator. The Laplacian operator for a nucleus has the form

(9) $$\Delta =\frac{1}{\sqrt{g}}\sum_{i,j}\frac{\partial }{\partial q_{i}}\sqrt{g}g_{ij}^{-1}\frac{\partial }{\partial q_{j}}$$

with *g* is the determinant of the matrix $g_{ij}$ and $g_{ij}^{-1}$ is the inverse of the matrix $g_{ij}$.

In the collective model of Bohr, for a gamma rigid nucleus model, particularly for axially symmetric prolate case, $\frac{d\gamma }{dt}=\overset{˙}{\gamma }=0$, and γ=0, then the classical kinetic energy reduces to the form given as [53](10a)
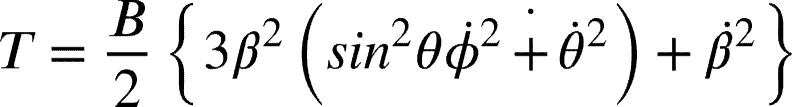
 It is clearly shown in equation (10a) that three degrees of freedom is used in the motion of the gamma rigid nucleus for axially symmetric prolate case. The generally coordinates are $x_{1}=\phi$, $x_{2}=\theta$, $x_{3}=\beta$, and the kinetic energy has a quadratic form written as [59]

(10b) $$T=\sum g_{ij}d{\overset{˙}{x}}_{i}d{\overset{˙}{x}}_{j}$$

where *x* is space of curve and $g_{ij}$ is a metric tensor.

From equation (a) and (b) the metric tensor is constructed as

(11) $$g_{ij}=\left(\begin{array}{ccc} 3\beta^{2}\sin^{2}\theta \; & 0\; & 0\\ 0\; & 3\beta^{2}\; & 0\\ 0\; & 0\; & 1 \end{array}\right)$$

In equation (11), *β* is variable in the radial direction. It corresponds to nucleus deformation, *ϕ* and *θ* are parts of Euler angles. From equation (11), the determinant of the matrix is

(12) $$g=9\beta^{2}\sin^{2}\theta$$

and the matrix inverse $g_{ij}$ is

(13) $$g_{ij}^{-1}=\left(\begin{array}{ccc} \frac{1}{3\beta^{2}\sin^{2}\theta }\; & 0\; & 0\\ 0\; & \frac{1}{3\beta^{2}}\; & 0\\ 0\; & 0\; & 1 \end{array}\right)$$

By inserting equations (12), (13) into equation (9), the Laplacian operator is obtained as follows

(14) $$\Delta =\frac{1}{\beta^{2}}\frac{\partial }{\partial \beta }\left(\beta^{2}\frac{\partial }{\partial \beta }\right)+\frac{1}{3\beta^{2}}\left[\frac{1}{\sin^{2}\theta }\frac{\partial^{2}}{\partial \varphi^{2}}+\frac{1}{\sin\theta }\frac{\partial }{\partial \theta }\left(\sin\theta \frac{\partial }{\partial \theta }\right)\right]$$

with $\left[\frac{1}{\sin^{2}\theta }\frac{\partial^{2}}{\partial \varphi^{2}}+\frac{1}{\sin\theta }\frac{\partial }{\partial \theta }\left(\sin\theta \frac{\partial }{\partial \theta }\right)\right]=\Delta_{\Omega }$ is Euler angle part of Bohr Mottelson Hamiltonian equation.

The Hamiltonian equation at higher energy is defined as [8]

(15) $$H=T+V\left(\beta \right)=\frac{P^{2}}{2B_{m}}+V\left(\beta \right)$$

with V(β) is the potential energy in *β* function, *P* is the operator of the momentum, and $B_{m}$ is the parameter of the mass. Equation (8) is inserted into equation (15), we get

(16) $$H=\frac{-\hbar^{2}\Delta }{2B_{m}}+\frac{\alpha \hbar^{4}\Delta^{2}}{B_{m}}+V\left(\beta \right)$$

or can be rewritten as

(17) $$\left\{\frac{-\hbar^{2}\Delta }{2B_{m}}+\frac{\alpha \hbar^{4}\Delta^{2}}{B_{m}}+V\left(\beta \right)-E\right\}\Psi \left(\beta ,\theta ,\phi \right)=0$$

To solve equation (17), the new wave function is introduced as follows

(18) $$\Psi \left(\beta ,\theta ,\phi \right)=\left(1+2\alpha_{\mathrm{ML}}\hbar^{2}\Delta \right)\chi \left(\beta ,\theta ,\phi \right)$$

By inserting equation (18) into equation (17), we have

(19) $$\left\{\frac{-\hbar^{2}\Delta }{2B_{m}}+\frac{\alpha \hbar^{4}\Delta^{2}}{B_{m}}+V\left(\beta \right)-E\right\}\left(1+2\alpha_{\mathrm{ML}}\hbar^{2}\Delta \right)\chi \left(\beta ,\theta ,\phi \right)=0$$

this equation reduces to

(20) $$\left\{\left(1+4\alpha_{\mathrm{ML}}B_{m}\left(E-V\left(\beta \right)\right)\right)\Delta +\frac{2B_{m}}{\hbar^{2}}\left(E-V\left(\beta \right)\right)\right\}\chi \left(\beta ,\theta ,\phi \right)=0$$

In this case, we have taken that $\alpha_{\mathrm{ML}}^{2}\approx 0$ since $\alpha_{\mathrm{ML}}$ is a small parameter, then equation (20) reduces to

(21) $$\Delta \chi \left(\beta ,\theta ,\phi \right)+\left[\frac{\frac{2B_{m}}{\hbar^{2}}E\left(1-\varepsilon V\left(\beta \right)\right)}{\left(1+4\alpha_{\mathrm{ML}}B_{m}E\right)\left(1-\frac{4\alpha_{\mathrm{ML}}B_{m}V\left(\beta \right)}{1+4\alpha_{\mathrm{ML}}B_{m}E}\right)}\right]\chi \left(\beta ,\theta ,\phi \right)=0$$

It is seen that the Schrodinger-like equation in equation (21) has an unfamiliar energy potential term in which equation (21) could not be solved analytically except for infinity well potential. In References [41], [43] the approximate solution of the Schrodinger-like equation in equation (21) is obtained by applying binomial expansion to the first degree of the potential term. In this work, this unfamiliar potential term will be manipulated by applying the q deformed hyperbolic potential concept and its spatial translation [44], [45] such that the potential term reduces to hyperbolic function potential. To solve equation (21), we set new parameters as

(22) $$\frac{2B_{m}}{\hbar^{2}}E=\omega ;\;1+4B_{m}\alpha_{\mathrm{ML}}E=\mu ;\frac{4B_{m}\alpha_{\mathrm{ML}}}{1+4B_{m}\alpha_{\mathrm{ML}}E}=-\tau ;E=\frac{1}{\varepsilon }$$

Such that equation (21) reduces to

(23) $$\Delta \chi \left(\beta ,\theta ,\phi \right)+\frac{\omega }{\mu }\left[\frac{\left(1-\varepsilon V\right)}{\left(1+\tau V\right)}\right]\chi \left(\beta ,\theta ,\phi \right)=0$$

By doing mathematical manipulation to the equation (23), then equation (23) is rewritten as

(24) $$\Delta \chi \left(\beta ,\theta ,\phi \right)+\frac{\omega }{\mu }\left[1+\frac{\left(-\tau -\varepsilon \right)V}{\left(1+\tau V\right)}\right]\chi \left(\beta ,\theta ,\phi \right)=0$$

By substituting equation (14) into equation (24), the Bohr Mottelson Hamiltonian equation for the certain energy potential V(β) is given by

(25) $$\left\{\begin{array}{c} \frac{1}{\beta^{2}}\frac{\partial }{\partial \beta }\left(\beta^{2}\frac{\partial }{\partial \beta }\right)\\ +\frac{1}{3\beta^{2}}\left[\begin{array}{c} \frac{1}{\sin\;\theta }\frac{\partial }{\partial \theta }\left(\sin\;\theta \frac{\partial }{\partial \theta }\right)\\ +\frac{1}{\sin^{2}\theta }\frac{\partial^{2}}{\partial \phi^{2}} \end{array}\right] \end{array}\right\}\chi \left(\beta ,\theta ,\phi \right)+\frac{\omega }{\mu }\left[1+\frac{\left(-\tau -\varepsilon \right)V}{\left(1+\tau V\right)}\right]\chi \left(\beta ,\theta ,\phi \right)=0$$

Equation (25) is solved by variable separation method by setting

(26) $$\chi \left(\beta ,\theta ,\phi \right)=\frac{R\left(\beta \right)}{\beta }P(\theta )\Phi (\phi )$$

and by inserting equation (26) into equation (25), the form of the equation to be

(27) $$\left\{\frac{3\beta^{2}}{R\beta^{2}}\frac{\partial }{\partial \beta }\left(\beta^{2}\frac{\partial }{\partial \beta }\left(\frac{R}{\beta }\right)\right)+\left[\begin{array}{c} \frac{1}{P\sin\;\theta }\frac{\partial }{\partial \theta }\left(\sin\;\theta \frac{\partial P}{\partial \theta }\right)\\ +\frac{1}{\Phi \sin^{2}\theta }\frac{\partial^{2}\Phi }{\partial \phi^{2}} \end{array}\right]\right\}+\frac{\omega }{\mu }\left[1+\frac{\left(-\tau -\varepsilon \right)V}{\left(1+\tau V\right)}\right]3\beta^{2}=0$$

From equation (27) we get the Bohr Mottelson Hamiltonian equation within the minimal length formalism for the *β* function given as

(28) $$\frac{\partial^{2}R}{\partial \beta^{2}}-\frac{\omega }{\mu }\frac{\left(\tau +\varepsilon \right)V}{\left(1+\tau V\right)}R+\frac{\omega }{\mu }R-\frac{\delta }{3\beta^{2}}R=0$$

and

(29) $$\frac{1}{P\sin\;\theta }\frac{\partial }{\partial \theta }\left(\sin\;\theta \frac{\partial P}{\partial \theta }\right)+\frac{1}{\Phi \sin^{2}\theta }\frac{\partial^{2}\Phi }{\partial \phi^{2}}=-\delta =-L(L+1)$$

for the angular part of the Bohr Mottelson Hamiltonian equation.

The angular part of Bohr Mottelson equation simply reduces to the usual associated Legendre equation as a function of (*θ*) and simple differential equation as a function of (*ϕ*), respectively given as

(30) $$\frac{1}{\sin\theta }\frac{\partial }{\partial \theta }\left(\sin\theta \frac{\partial P}{\partial \theta }\right)+L(L+1)P-\frac{m^{2}}{\sin^{2}\theta }P=0$$

and

(31) $$\frac{\partial^{2}\Phi }{\partial \phi^{2}}+m^{2}\Phi =0$$

Equation (30) is a second-order differential equation of associated Legendre function. It is reduces to the second-order differential equation of Legendre function for m = 0 given as

(32) $$\frac{1}{\sin\theta }\frac{\partial }{\partial \theta }\left(\sin\theta \frac{\partial P}{\partial \theta }\right)+L(L+1)P=0$$

Equation (32) is solved by setting a new variable given as

(33) $$\cos\theta =w;\;\sin\theta =\sqrt{1-\cos^{2}\theta }=\sqrt{1-w^{2}}$$

then we have

(34) $$\frac{d}{d\theta }=-\sin\theta \frac{d}{dw}\text{ and}\;\frac{d^{2}}{d\theta^{2}}=-w\frac{d}{dw}+\left(1-w^{2}\right)\frac{d^{2}}{dw^{2}}$$

Substituting the relationships in (33), (34) into equation (32), we get

(35) $$\left(1-w^{2}\right)\frac{d^{2}P\left(w\right)}{dw^{2}}-2w\frac{dP\left(w\right)}{dw}+L(L+1)P\left(w\right)=0$$

Equation (35) is solved in the form of Legendre polynomial by using Legendre Polynomial generating function [60] given as

(36) $$g\left(t,w\right)=\frac{1}{{\left(1-2wt+t^{2}\right)}^{1/2}}=\sum_{n=0}^{\infty }P_{n}(w)t^{n}$$

By differentiating equation (36) respect to *t* we get

(37) $$\frac{w-t}{{\left(1-2wt+t^{2}\right)}^{3/2}}=\sum_{n=0}^{\infty }nP_{n}(w)t^{n-1}$$

By inserting equation (36) into equation (37) we obtain

(38) $$\left(t-w\right)\sum_{n=0}^{\infty }P_{n}\left(w\right)t^{n}+\left(1-2wt+t^{2}\right)\sum_{n=0}^{\infty }nP_{n}\left(w\right)t^{n-1}=0$$

that could be rewritten by using distinctive summation indices as

(39) $$\;\sum_{n=0}^{\infty }P_{m}\left(w\right)t^{m+1}-\sum_{n=0}^{\infty }wP_{n}\left(w\right)t^{n}+\sum_{n=0}^{\infty }sP_{s}\left(w\right)t^{s-1}-\sum_{n=0}^{\infty }2wnP_{n}\left(w\right)t^{n}+\sum_{n=0}^{\infty }mP_{m}\left(w\right)t^{m+1}=0$$

By letting m=n−1;s=n+1; then from equation (39) we obtain a recurrence relation given as

(40) $$(2n+1)w\;P_{n}\left(w\right)={\mathrm{nP}}_{n-1}\left(w\right)+(n+1)P_{n+1}\left(w\right)$$

with n = 0, 1, 2, …. In the same way, by differentiating equation (36) respect to *w* we get another recurrence relations given as

(41) $$P_{n+1}^{\prime }\left(w\right)+P_{n-1}^{\prime }\left(w\right)=2wP_{n}^{\prime }(w)+P_{n}\left(w\right)$$

By knowing the lowest values of $P_{n-1}\left(w\right)$ and $P_{n}\left(w\right)$ for n = 1, in the recurrence relation in equation (40), then the value of the higher Legendre Polynomial, $P_{2}\left(w\right),P_{3}\left(w\right),\;\ldots$ will be constructed easily. By using simple mathematical manipulation, from 2 recurrence relations in equations (40) and (41) we obtain the linear second-order differential equation of Legendre function as expressed in equation (35).

By applying binomial theorem, the Legendre polynomial generating function in equation (36) could be expanded into the form

(42) $$\frac{1}{{\left(1-2wt+t^{2}\right)}^{1/2}}=\sum_{n=1}^{\infty }\frac{\left(2n\right)!}{2^{2n}{\left(n!\right)}^{2}}{\left(2wt-t^{2}\right)}^{n}$$

Moreover, by expanding using binomial theorem to the last term in equation (42) we get

(43) $$\frac{1}{{\left(1-2wt+t^{2}\right)}^{1/2}}=\sum_{n=1}^{\infty }\sum_{k=0}^{n}{\left(-1\right)}^{k}\frac{\left(2n\right)!}{2^{2n}n!k!(n-k)!}{\left(2w\right)}^{n-k}t^{n+k}$$

By re arranging the summation in equation (43) and taking n+k→n then equation (43) reduces to

(44) $$\frac{1}{{\left(1-2wt+t^{2}\right)}^{1/2}}=\sum_{n=1}^{\infty }\sum_{k=0}^{n/2}{\left(-1\right)}^{k}\frac{\left(2n-2k\right)!}{2^{2n-2k}(n-k)!k!(n-2k)!}{\left(2w\right)}^{n-2k}t^{n}$$

By comparing equation (36) with equation (44) we have

(45) $$P_{n}\left(w\right)=\sum_{k=0}^{n/2}{\left(-1\right)}^{k}\frac{\left(2n-2k\right)!}{2^{2n-2k}(n-k)!k!(n-2k)!}{\left(2w\right)}^{n-2k}$$

and finally we obtain the Legendre polynomial that is obtained from equation (45) given as

(46) $$P_{n}\left(w\right)=\frac{1}{2^{n}n!}\frac{d^{n}}{dw^{n}}{\left(w^{2}-1\right)}^{2}$$

with n = 0, 1, 2, 3,… and usually n symbol is renamed by *l*.

In the new variable, the second differential equation of associated Legendre function expressed in equation (30) is given as

(47) $$\left(1-w^{2}\right)\frac{d^{2}P(w)}{dw^{2}}-2w\frac{dP\left(w\right)}{dw}+L\left(L+1\right)P\left(w\right)-\frac{m^{2}}{1-w^{2}}P\left(w\right)=0$$

The solution of equation (47) is obtained by deriving equation (35) concerning *w* m times and by renaming P(w) as y(w) we have

(48) $$\frac{d^{m}}{dw^{m}}\left[\left(1-w^{2}\right)\frac{d^{2}y\left(w\right)}{dw^{2}}-2w\frac{dy\left(w\right)}{dw}+\left[L(L+1)\right]y\left(w\right)\right]=0$$

with the help of Leibnitz's formula defined as

(49) $$\frac{d^{n}}{dx^{n}}\left[A(x)B(x)\right]=\sum_{s-0}^{n}\left({}_{s}^{n}\right)\frac{d^{n-s}}{dx^{n-s}}A(x)\frac{d^{s}}{dx^{s}}B(x),\left({}_{s}^{n}\right)=\frac{n!}{\left(n-s\right)!s!}$$

then equation (48) becomes to

(50) $$\left(1-w^{2}\right)u^{\prime \prime }-2w\left(m+1\right)u^{\prime }-\left(m^{2}+m\right)u+\left[L(L+1)\right]u=0$$

where $\frac{d^{m}y(w)}{dw^{m}}=u(w)$ and $u^{\prime \prime }=\frac{d^{2}u\left(w\right)}{dw^{2}}$.

By introducing a new function v(w) in equation (50)

(51) $$v\left(w\right)=u(w){\left(1-w^{2}\right)}^{-m/2}$$

then equation (50) reduces to

(52) $$\left(1-w^{2}\right)v^{\prime \prime }-2wv^{\prime }+\left[L\left(L+1\right)-\frac{m^{2}}{1-w^{2}}\right]v=0$$

By comparing equation (47) and (52) we conclude that v(w) corresponds to the associated Legendre function, therefore the associated Legendre Polynomial,

(53) $$P_{L}^{m}\left(w\right)=v\left(w\right)={\left(1-w^{2}\right)}^{-\frac{m}{2}}u\left(w\right)={\left(1-w^{2}\right)}^{-\frac{m}{2}}\frac{d^{m}P_{L}(w)}{dw^{m}}$$

with $P_{L}(w)$ is the same with $P_{n}(w)$ expressed in equation (46).

## 3 Woods-Saxon potential

The Woods Saxon potential has an important role in nuclear and microscopic physics, it is used to describe the interaction between neutron and nucleus [61], [62], [63]. The Woods Saxon potential could be used to measure the nuclear size and to determine the diffuseness of the nuclear surface [51], [64]. According to Capak et al. [25], the potential can describe well the ground state and $\gamma_{1}$ bands of many prolate deformed nuclei. But, it fails in describing the $\beta_{1}$ bands due to its lack of a hardcore. The Woods Saxon potential [28], [29], [64] in general form appears as

(54) $$V\left(\beta \right)=-\frac{V_{0}}{1+e^{\frac{\beta -\beta_{0}}{r_{0}}}}$$

with $\beta_{0}$ is the range of potential, $r_{0}$ is the diffusivity of nuclear surface, and $V_{0}$ is the depth of potential. The potential in equation (54) could be simplified by setting

(55) $$e^{\frac{-\beta_{0}}{r_{0}}}=\frac{1}{\sigma }$$

then, the Woods-Saxon potential turns to the following form

(56) $$V\left(\beta \right)=-\frac{\sigma V_{0}e^{-\frac{\beta }{2r_{0}}}}{\left(\sigma e^{-\frac{\beta }{2r_{0}}}+e^{\frac{\beta }{2r_{0}}}\right)}$$

The Woods-Saxon potential that is expressed in equation (56) could be considered as deformed hyperbolic potential with the deformed parameter *σ*. The deformed hyperbolic potential was proposed by Arai some years ago [44].

The applications of deformed potential have been investigated by Ikhdair [65] and Dutra [45]. The formalism of q deformed hyperbolic functions is given as

(57) $$\sinh_{q}\in r=\frac{e^{\in r}-qe^{-\in r}}{2};\cosh_{q}\in r=\frac{e^{\in r}+qe^{-\in r}}{2}$$

(58) $$\tanh_{q}\in r=\frac{\sinh_{q}\in r}{\cosh_{q}\in r};\cosh_{q}^{2}\in r-\sinh_{q}^{2}\in r=q$$

The deformed potentials can be transformed into the non-deformed potentials by a convenient translation of a spatial variable that was proposed by Dutra [45], which is

(59) $$r\to r+\frac{\ln\sqrt{q}}{\in }$$

Then, by substituting equation (59) into equation (57), the relation of the q deformed hyperbolic function to the usual hyperbolic function is

(60) $$\sinh_{q}\in r=\sqrt{q}\sinh\in r;\cosh_{q}\in r=\sqrt{q}\cosh\in r$$

The concept of q deformed hyperbolic potential and translation of spatial variable is applied to manipulate the potential term due to the presence of minimal length in Bohr Mottelson equation such that it could be solved analytically.

## 4 Thermodynamic properties

In the classical limit, the vibrational mean energy, the vibrational specific heat, the vibrational free energy, and the vibrational entropy are obtained from the energy spectra equation. These thermodynamical functions [46], [47], [48], [49] are defined as follows

(1) The vibrational mean energy (U)
  (61) $$U\left(\beta^{\prime }\right)=-\frac{\partial }{\partial \beta^{\prime }}\ln Z_{vib}\left(\beta^{\prime }\right)$$
(2) The vibrational specific heat (C)
  (62) $$C\left(\beta^{\prime }\right)=\frac{\partial }{\partial T}U=-k\beta^{\prime \;2}\frac{\partial }{\partial \beta^{\prime }}U$$
(3) The vibrational free energy (F)
  (63) $$F\left(\beta^{\prime }\right)=-kT\ln Z_{vib}\left(\beta^{\prime }\right)$$
(4) The vibrational entropy (S)
  (64) $$S\left(\beta^{\prime }\right)=k\ln Z_{vib}\left(\beta^{\prime }\right)+kT\frac{\partial }{\partial T}\ln Z_{vib}\left(\beta^{\prime }\right)$$

The $Z_{vib}$ which is the vibrational partition function that can be determined by direct summation over all possible vibrational energy levels, given as [46], [47], [48]

(65) $$Z_{vib}\left(\beta^{\prime }\right)=\sum_{n=0}^{n_{\max}}e^{-\beta^{\prime }E_{n}}$$

where $\beta^{\prime }=\frac{1}{kT}$, *T* is the temperature, $E_{n}$ is the energy levels of system quantum for Woods Saxon potential, and *k* is the Boltzmann's constant. Meanwhile, $n=0,1,2,3,...n_{\max}$, $n_{\max}$ denotes the upper bound vibration quantum number. The maximum value $n_{\max}$ is obtained from a condition that [49]

(66) $$\frac{dE_{n}}{dn}=0$$

Those thermodynamic properties that are presented in equations (65-66) are obtained from the energy eigenvalue equation of the quantum system and are expressed in terms of the imaginary error functions. In mathematics, the imaginary error function is known to be [46], [47], [48], [49]

(67) erfi(x)=−ierf(ix)

which is presented in integral representation as

(68) $$\mathrm{erfi}\left(x\right)=\frac{2}{\sqrt{\pi }}\int_{0}^{x}e^{t^{2}}dt$$

and

(69) $$\mathrm{erf}\left(x\right)=\frac{2}{\sqrt{\pi }}\int_{0}^{x}e^{-t^{2}}dt$$

for the error function.

## 5 Hypergeometric method

The term of Hypergeometric function which is expressed by the second-order differential equation is given as [10], [65]

(70) $$z\left(1-z\right)\frac{d^{2}\phi }{dz^{2}}+\left(c^{\prime }-\left(a^{\prime }+b^{\prime }+1\right)z\right)\frac{d\phi }{dz}-a^{\prime }b^{\prime }\phi =0$$

Equation (66) has the following solution [1], [63]

(71) $$\phi_{1}={}_{2}F_{1}\left(a^{\prime };b^{\prime };c^{\prime };z\right)=\sum_{n=0}\frac{{\left(a^{\prime }\right)}_{n}{\left(b^{\prime }\right)}_{n}}{{\left(c^{\prime }\right)}_{n}n!}z^{n}$$

with

(72) $$\sum_{n=0}\frac{{\left(a^{\prime }\right)}_{n}{\left(b^{\prime }\right)}_{n}}{{\left(c^{\prime }\right)}_{n}n!}z^{n}=1+\frac{a^{\prime }b^{\prime }}{c^{\prime }}z+\frac{a^{\prime }\left(a^{\prime }+1\right)\left(b^{\prime }\right)\left(b^{\prime }+1\right)}{c^{\prime }\left(c^{\prime }+1\right)}\frac{z^{2}}{2!}+\frac{a^{\prime }\left(a^{\prime }+1\right)\left(a^{\prime }+2\right)\left(b^{\prime }\right)\left(b^{\prime }+1\right)\left(b^{\prime }+2\right)}{c^{\prime }\left(c^{\prime }+1\right)\left(c^{\prime }+2\right)}\frac{z^{3}}{3!}+...$$

The energy eigenvalue is obtained from the specific condition taken from equation (70) that is [10],

(73) $$a^{\prime }=-n;b^{\prime }=-n$$

With n is the radial quantum number, where n=1,2,3,.... By applying the suitable variable and wave function substitutions then the one dimensional Schrodinger equation reduces to standard hypergeometric equation expressed in equation (70) and from this equation, the energy spectra and the wave function are obtained.

## 6 Results and discussion

### 6.1 Bohr Mottelson Hamiltonian equation for rigid nucleus, γ=0 with minimal length effect for Woods-Saxon potential

By inserting equation (56) into equation (28), the radial part of Bohr Mottelson Hamiltonian equation within the minimal length formalism changes in the following condition

(74) $$\frac{\partial^{2}R}{\partial \beta^{2}}-\frac{\omega }{\mu }\frac{\left(\tau +\varepsilon \right)\left(-\frac{\sigma V_{0}e^{-\frac{\beta }{2r_{0}}}}{\left(\sigma e^{-\frac{\beta }{2r_{0}}}+e^{\frac{\beta }{2r_{0}}}\right)}\right)}{\left(1+\tau \left(-\frac{\sigma V_{0}e^{-\frac{\beta }{2r_{0}}}}{\left(\sigma e^{-\frac{\beta }{2r_{0}}}+e^{\frac{\beta }{2r_{0}}}\right)}\right)\right)}R+\frac{\omega }{\mu }R-\frac{\delta }{3\beta^{2}}R=0$$

Equation (74) can be rewritten as

(75) $$\frac{\partial^{2}R}{\partial \beta^{2}}-\frac{\omega }{\mu }\frac{\left(\tau +\varepsilon \right)\left(-\sigma V_{0}e^{-\frac{\beta }{2r_{0}}}\right)}{\left(e^{\frac{\beta }{2r_{0}}}-\sigma \left(\tau V_{0}-1\right)e^{-\frac{\beta }{2r_{0}}}\right)}R+\frac{\omega }{\mu }R-\frac{\delta }{3\beta^{2}}R=0$$

By setting new parameters in equation (75) as

(76) $$\frac{1}{2r_{0}}=\gamma^{\prime };\;\;\sigma \left(\tau V_{0}-1\right)=q;$$

and by inserting equations (57), (60) and (76) into equation (75) then we have

(77) $$\frac{\partial^{2}R}{\partial \beta^{2}}+\frac{\omega }{\mu }\frac{\left(\tau +\varepsilon \right)\sigma V_{0}}{\left(2q\right)}\left(\coth\gamma^{\prime }\beta -1\right)R+\frac{\omega }{\mu }R-\frac{\delta \gamma^{\prime \;2}}{3\sinh^{2}\gamma^{\prime }\beta }R=0$$

that yields

(78) $$\frac{\partial^{2}R}{\partial \beta^{2}}-\left(\frac{\gamma^{\prime \;2}L^{\prime }\left(L^{\prime }-1\right)}{\sinh^{2}\gamma^{\prime }\beta }-2\eta \left(\coth\gamma^{\prime }\beta \right)-E^{\prime }\right)R=0$$

with

(79) $$\frac{\delta }{3}=\frac{L(L+1)}{3}=L^{\prime }(L^{\prime }-1);\;\frac{\omega }{\mu }\frac{\left(\tau +\varepsilon \right)\sigma V_{0}}{\left(2q\right)}=2\eta ;\;-\frac{\omega }{\mu }\frac{\left(\tau +\varepsilon \right)\sigma V_{0}}{\left(2q\right)}+\frac{\omega }{\mu }=E^{\prime }$$

and the approximate value of $\frac{1}{\beta^{2}}$ [24] is given as

(80) $$\frac{1}{\beta^{2}}=\frac{\gamma^{\prime \;2}}{\sinh^{2}\gamma^{\prime }\beta }$$

Equation (78) is the radial part of the Bohr Mottelson Hamiltonian equation for Woods-Saxon potential within the minimal length formalism that had reduced to one dimensional Schrodinger-like equation that can be solved by the hypergeometric method. To solve equation (78), we should set a new variable that is

(81) $$\coth\gamma^{\prime }\beta =1-2z$$

$$\frac{d}{d\beta }=\frac{d}{dz}\frac{dz}{d\beta }=2\gamma^{\prime }\left(z^{2}-z\right)\frac{d}{dz}$$

(82) $$\frac{d^{2}}{d\beta^{2}}=4\gamma^{\prime \;2}z^{2}{\left(z-1\right)}^{2}\frac{d^{2}}{dz^{2}}+4\gamma^{\prime \;2}z\left(z-1\right)\left(2z-1\right)\frac{d}{dz}$$

then equation (78) reduces to

(83) $$4\gamma^{\prime \;2}z^{2}{\left(z-1\right)}^{2}\frac{d^{2}R\left(\beta \right)}{dz^{2}}+4\gamma^{\prime \;2}z\left(z-1\right)\left(2z-1\right)\frac{dR\left(\beta \right)}{dz}-\left[\begin{array}{c} 4\gamma^{\prime \;2}L^{\prime }\left(L^{\prime }-1\right)z\left(z-1\right)-\\ 2\eta \left(1-2z\right)-E^{\prime } \end{array}\right]R\left(\beta \right)=0$$

For mathematical simplicity, we rename the parameters in equation (83) into

(84) $$\frac{\eta }{\gamma^{\prime \;2}}=\eta^{\prime };\;-\frac{E^{\prime }}{\gamma^{\prime \;2}}=k^{2}$$

then equation (83) reduces to

(85) $$z\left(1-z\right)\frac{d^{2}R}{dz^{2}}+\left(1-2z\right)\frac{dR}{dz}+\left[L^{\prime }\left(L^{\prime }-1\right)-\frac{-2\eta^{\prime }+k^{2}}{4z}-\frac{2\eta^{\prime }+k^{2}}{4\left(1-z\right)}\right]R=0$$

To apply the hypergeometric method, we set a new wave function as follows

(86) $$R\left(\beta \right)=z^{\alpha }{\left(1-z\right)}^{\vartheta }f\left(z\right)$$

and set new parameters as

(87) $$-2\eta^{\prime }+k^{2}=4\alpha^{2};\;2\eta^{\prime }+k^{2}=4\vartheta^{2}$$

The hypergeometric differential equation is obtained by using equations (85), (86) and (87) given as

(88) $$z\left(1-z\right)f^{\prime \prime }\left(z\right)+\left[\left(2\alpha +1\right)-\left(2\alpha +2\vartheta +2\right)z\right]f^{\prime }\left(z\right)-\left[\begin{array}{c} \left(\alpha +\vartheta \right)\left(\alpha +\vartheta +1\right)\\ -L^{\prime }\left(L^{\prime }-1\right) \end{array}\right]f\left(z\right)=0$$

By comparing equations (70) and (88), we obtain

(89) $$a^{\prime }=\alpha +\vartheta -L^{\prime }+1;\;b^{\prime }=\alpha +\vartheta +L^{\prime };\;c^{\prime }=2\alpha +1$$

Since we take $a^{\prime }=-n$, then from equation (89) we obtain

(90) $$\alpha +\vartheta =L^{\prime }-1-n\to \alpha^{2}+2\alpha \vartheta +\vartheta^{2}={\left(L^{\prime }-1-n\right)}^{2}$$

Therefore, the energy spectra that are obtained from equation (87), (88), and (90) is given as

(91) $$k^{2}={\left(L^{\prime }-1-n\right)}^{2}+\frac{\eta^{\prime \;2}}{{\left(L^{\prime }-1-n\right)}^{2}}$$

By using equations (79), (84), and (91), we obtain

(92) $$\frac{\omega }{\gamma^{\prime \;2}\mu }\left(\frac{\left(\tau +\varepsilon \right)\sigma V}{2q}-1\right)={\left(L^{\prime }-1-n\right)}^{2}+\left[\frac{\frac{\omega }{\gamma^{\prime \;2}\mu }{\left(\frac{\left(\tau +\varepsilon \right)\sigma V}{2q}\right)}^{2}}{{\left(L^{\prime }-1-n\right)}^{2}}\right]=0$$

By inserting equation (22) into equation (92), we get the energy spectra equation given as

(93) $$\frac{\frac{2B_{m}}{\hbar^{2}}E}{\gamma^{\prime \;2}\left(1+4B_{m}\alpha_{\mathrm{ML}}E\right)}\left[\frac{\left(\frac{1}{E}-\frac{4B_{m}\alpha_{\mathrm{ML}}}{1+4B_{m}\alpha_{\mathrm{ML}}E}\right)\sigma V_{0}}{2q}-1\right]=\left[\begin{array}{c} {\left(L^{\prime }-1-n\right)}^{2}+\\ \frac{{\left(\frac{\frac{2B_{m}}{\hbar^{2}}E}{\gamma^{\prime \;2}\left(1+4B_{m}\alpha_{\mathrm{ML}}E\right)}\frac{\left(\frac{1}{E}-\frac{4B_{m}\alpha_{\mathrm{ML}}}{1+4B_{m}\alpha_{\mathrm{ML}}E}\right)\sigma V_{0}}{4q}\right)}^{2}}{{\left(L^{\prime }-1-n\right)}^{2}} \end{array}\right]$$

The energy equation in equation (93) is rewritten as

(94) $$E_{n}=-\frac{4\gamma^{\prime \;2}\left(1+4B_{m}\alpha_{\mathrm{ML}}E\right)}{\frac{2B_{m}}{\hbar^{2}}}\left[\begin{array}{c} \frac{{\left(n+1-L^{\prime }\right)}^{2}}{4}+\frac{{\left(\frac{{\left(\frac{2B_{m}}{\hbar^{2}}\sigma V_{0}\right)}^{2}}{\gamma^{\prime \;4}\left(4\right)\left(16q^{2}\right){\left(1+4B_{m}\alpha_{\mathrm{ML}}E\right)}^{4}}\right)}^{2}}{{\left(n+1-L^{\prime }\right)}^{2}}-\\ \frac{2B_{m}\sigma V_{0}}{2\left(4q\right){\left(1+4B_{m}\alpha_{\mathrm{ML}}E\right)}^{2}} \end{array}\right]$$

or

(95) $$E_{n}=-\frac{4\gamma^{\prime \;2}\left(1+4B_{m}\alpha_{\mathrm{ML}}E\right)}{\frac{2B_{m}}{\hbar^{2}}}{\left[\frac{\left(n+1-L^{\prime }\right)}{2}+\frac{\left(\frac{{\left(\frac{B_{m}}{\hbar^{2}}\sigma V_{0}\right)}^{2}}{4\gamma^{\prime \;4}q{\left(1+4B_{m}\alpha_{\mathrm{ML}}E\right)}^{2}}\right)}{\left(n+1-L^{\prime }\right)}\right]}^{2}$$

with $L^{\prime }=\frac{1}{2}+\sqrt{\frac{L\left(L+1\right)}{3}+\frac{1}{4}}$.

The equation (95) is the energy spectra equation of the Bohr Mottelson Hamiltonian for Woods-Saxon potential with minimal length effect for rigid γ=0. The energy spectra equation which is expressed in equation (95) can not be calculated analytically, therefore the energy spectra are calculated numerically by Matlab software for various of $n,\alpha_{\mathrm{ML}}$, and *L* which are the radial quantum number, the minimal length parameter, and the angular quantum number. Parameter values used in the numerical calculation of energy spectra are presented in Table 1.

**Table 1:** Constant values for Woods-Saxon potential and energy spectra.

Parameters	Values
Depth of potential (*V*_0_)	(40.5+0.13A)MeV
Range of potential (*β*_0_)	$\left(1.285A^{\frac{1}{3}}\right)\;\text{fm}$
Diffusivity of nuclear surface (*r*_0_)	0.65 fm
Planck's constant (*ħ*)	6.582 × 10^−22^ MeV.s
Speed of light (*c*)	2.997 × 10^−23^ fm/s
Boltzmann's constant (*k*)	8.617 × 10^−11^ MeV/K
Atomic mass number (*A*)	48u
	50u
	52u
	54u

According to Perey's investigation [66], those values of parameters are fit to the medium weight of nuclei in the mass range of ^45^Sc to ^76^Ge. So, in this work, we choose the atomic mass numbers 48, 50, 52, and 54. The results of the energy spectra of these three atoms are shown in Tables 2, 3, 4, 5, 6, 7 and 8.

**Table 2:** The energy spectra of Woods-Saxon potential for various L with *α*_*ML*_ = 0 and n=2 for different atoms.

L	E (MeV)			
	A=48	A=50	A=52	A=54
2	-992.364580	-992.495028	-992.626932	-992.758465
4	-48.081109	-48.304022	-48.528917	-48.752795
6	-1598.242277	-1598.372087	-1598.504748	-1598.635260
7	-3336.901841	-3337.032763	-3337.162635	-3337.292459
11	-16726.037182	-16726.167951	-16726.297614	-16726.427170
14	-33538.205295	-33538.335282	-33538.465948	-33538.595294

**Table 3:** The energy spectra of Woods-Saxon potential for various L with *α*_*ML*_ = 0 and n=3 for different atoms.

L	E (MeV)			
	A=48	A=50	A=52	A=54
4	-756.956618	-757.088447	-757.220043	-757.353406
6	-98.462502	-98.613679	-98.764295	-98.915416
7	-722.458973	-722.590096	-722.723673	-722.855701
11	-9649.428957	-9649.558466	-9649.689663	-9649.819548
14	-23110.416434	-23110.546045	-23110.676128	-23110.806456

**Table 4:** The energy spectra of Woods-Saxon potential for various L with *α*_*ML*_ = 0 and n=4 for different atoms.

L	E (MeV)			
	A=48	A=50	A=52	A=54
4	-3411.547924	-3411.677879	-3411.808499	-3411.938802
6	-533.009570	-533.142958	-533.275880	-533.408337
7	-51.576784	-51.775607	-51.975270	-52.175087
11	-4510.531271	-4510.661331	-4510.792076	-4510.922507
14	-14620.329616	-14620.460175	-14620.590552	-14620.720744

**Table 5:** The energy spectra of Woods-Saxon potential for various L with *α*_*ML*_ = 0 and n=6 for different atoms.

L	E (MeV)			
	A=48	A=50	A=52	A=54
4	-14534.091650	-14534.221150	-14534.352877	-14534.482829
6	-7218.149589	-7218.279663	-7218.409297	-7218.540490
7	-4504.708035	-4504.839843	-4504.969810	-4505.099935
11	-51.872645	-52.070412	-52.268099	-52.467439
14	-3453.283808	-3453.413686	-3453.544037	-3453.675861

**Table 6:** The energy spectra of Woods-Saxon potential for various L with *α*_*ML*_ = 0 and n=8 for different atoms.

L	E (MeV)			
	A=48	A=50	A=52	A=54
4	-33407.465421	-33407.595646	-33407.725425	-33407.856759
6	-21654.329696	-21654.459085	-21654.589545	-27222.737075
7	-16714.801139	-16714.931927	-16715.061014	-16715.191400
11	-3331.898118	-3332.028560	-3332.159526	-3332.389801
14	-47.013191	-47.255145	-47.498844	-47.741287
16	-2540.634213	-2540.765807	-2540.895764	-2541.026086

**Table 7:** The energy spectra of Woods-Saxon potential for various *α*_*ML*_ with n=2, 3 and L=4, 6 for different atoms.

n	L	q	*α*_*ML*_	E (MeV)			
				A=48	A=50	A=52	A=54
2	4	3.6; 5.1; 6.9; 8.9	0.017	-48.080747	-48.303662	-48.527559	-48.751439
		1.3; 2.1; 4.1; 5.9	0.018	-48.080726	-48.303641	-48.527538	-48.751418
		0.6; 0.3; 1.6; 3.2	0.019	-48.080705	-48.303620	-48.527517	-48.751397
		-5.7;-5.1; -4.4; -3.5	0.022	-48.079967	-48.303599	-48.527517	-48.751376
3	6	7.1; 11.3; 16.1; 21	0.000481	-98.462502	-98.612646	-98.763496	-98.914259
		4.1; 7.9; 12.4; 17	0.000482	-98.462466	-98.612610	-98.763194	-98.914223
		1.1; 4.5; 8.7; 13	0.000483	-98.462430	-98.612574	-98.763158	-98.914187
		-7.9;-5.3; -2.1; 1.6	0.000486	-98.462322	-98.612466	-98.763112	-98.914124

**Table 8:** The energy spectra of Woods-Saxon potential for various *α*_*ML*_ with n=4, 6, 8 and L=7, 11, 14 for different atoms.

n	L	q	*α*_*ML*_	E (MeV)			
				A=48	A=50	A=52	A=54
4	7	7.5; 10.2; 13.2; 16	0.0049	-51.575835	-51.774061	-51.974069	-52.174859
		4.6; 7; 9.7; 12.8	0.0050	-51.575794	-51.774020	-51.974028	-52.174818
		1.8; 3.9; 6.3; 9.2	0.0051	-51.575753	-51.773979	-51.973987	-52.174776
		-5.7;-4.4; -2.8; -0.9	0.0054	-51.575630	-51.773856	-51.973863	-52.174653
6	11	8.6; 11.4; 14.6; 18	0.0046	-51.871501	-52.069164	-52.267140	-52.466430
		5.3; 7.7; 10.6; 13.8	0.0047	-51.871458	-52.069121	-52.267098	-52.466387
		2.1; 4.3; 6.8; 9.7	0.0048	-51.871416	-52.069079	-52.267055	-52.466344
		-6.4;-5.1; -3.5; -1.6	0.0051	-51.871288	-52.068951	-52.266927	-52.466217
8	14	2.3; 3.1; 4.1; 5.1	0.07	-47.012649	-47.254346	-47.497218	-47.740264
		1.1; 1.8; 2.5; 3.4	0.08	-47.012639	-47.254336	-47.497208	-47.740254
		0.1; 0.7; 1.3; 2.1	0.09	-47.012629	-47.254327	-47.497199	-47.740245
		-1.8;-1.4; -0.9; -0.4	0.12	-47.012599	-47.254297	-47.497170	-47.740218

**Figure 1:**
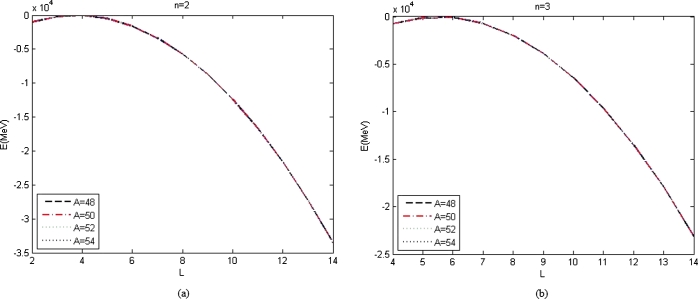
The graph of energy spectra listed in Tables 2, 3 as a function of *L* with *α*_*ML*_ = 0 for different atoms and (a) n=2, (b) n=3.

**Figure 2:**
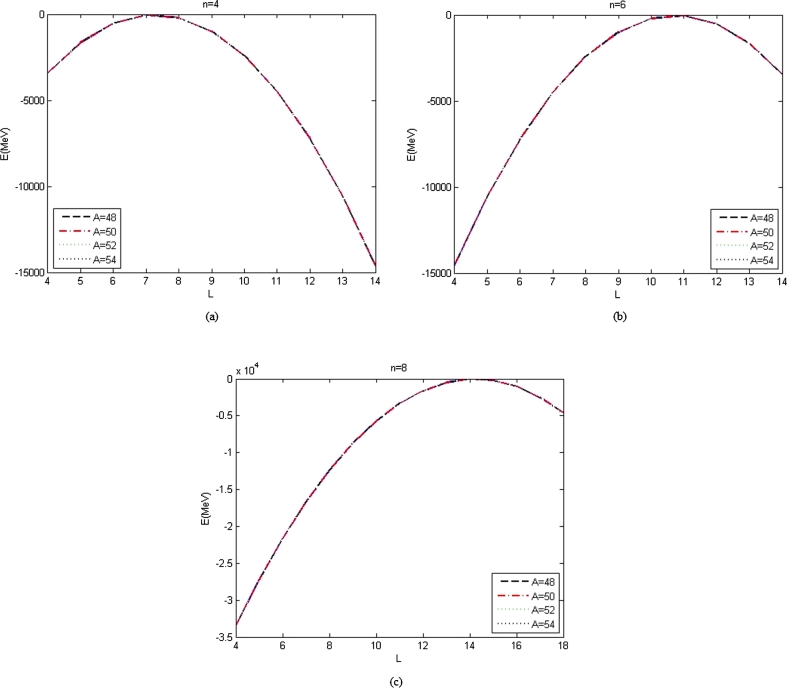
The graph of energy spectra listed in Tables 4, 5 and 6 as a function of *L* with *α*_*ML*_ = 0 for different atoms and (a) n=4, (b) n=6, (c) n=8.

The energy spectra that are calculated numerically for various n, L, and $\alpha_{\mathrm{ML}}$ are shown in Tables 2, 3, 4, 5, 6, 7 and 8. Fig. 1 shows the graphs of energy spectra of the four atoms without the presence of minimal length, $\alpha_{\mathrm{ML}}=0$ for various radial quantum numbers, (a) for n=2 and (b) for n = 3. The calculated energy spectra of the atoms with $\alpha_{\mathrm{ML}}=0$ are shown in Tables 2, 3, 4, 5 and 6 and the corresponding graphs are shown in Figs. 1, 2.

The energy spectra with $\alpha_{\mathrm{ML}}=0$ listed in Tables 2, 3, 4, 5 and 6 are plotted as a function of L for constant *n*, are shown in Figs. 1, 2. Figs. 1(a) and 1(b) show the graph of energy spectra listed in Tables 2, 3 as a function of the angular quantum number and the atomic mass for the constant values of radial quantum number, n=2 and n=3 and constant values of parameters which presented in Table 1. Figs. 2(a), 2(b), and 2(c) show the graph of energy spectra listed in Tables 4, 5 and 6 as a function of the angular quantum number and the atomic mass for constant values of parameters which presented in Table 1 and the constant values of radial quantum number, n=4, n=6, and n=8 for Figs. 2(a), 2(b), and 2(c), respectively. Figs. 1, 2 show that the graph of energy without the effect of minimal length, the maximum values of the energy of each value of n depend on the values of radial and orbital quantum numbers n and L. The greater the value of n, the maximum value of the energy is shifted toward the greater value of L. For small n, n=2, 3, the maximum value of energy is at lower L, and the maximum value of the energy is at higher L for big n, n = 6, 8. It is seen from Tables 2, 3, 4, 5 and 6 that the values of n and L that are chosen to be used to calculate energy spectra with the positive value of q are the same as the values of n and L for the maximum values of the energy spectra. The values of the minimal length parameter that are chosen to be used to calculate the energy spectra are also small. The maximum values of the energy spectra without minimal length are similar to the energy spectra with minimal length at the same n and L.

The energy spectra for four atoms with the effect of minimal length are shown in Tables 7, 8. It is seen from Tables 2, 3, 4, 5, 6, 7 and 8 that the values of radial and orbital quantum numbers that are used to calculate the energy numerically are very limited because there is a requirement that the values of q, the deformed potential parameters that be used to manipulate the unusual potential have to be positive. The values of q depend on the atomic mass, minimal length parameter, diffusivity, and the nucleus energy. Tables 7, 8 also shown that the minimal length parameter has the most effect on the energy spectra. The larger is the minimal length values, the higher is the energy spectra of the atoms.

It can be seen from Tables 2, 3, 4, 5 and 6 and Tables 7, 8 that the values of the energy spectra for the similar value of n and L but different values of minimal length are the same. The energy spectra with and without the minimal length are similar.

On the other hand, the Woods-Saxon potential is a simple one-body potential where it provides a model for the properties of bound-state and continuum single-particle wave functions. At this point, a high degree of accuracy is not expected from the calculations and the potential cannot be used for the total binding energy since it is not based upon a specific two-body interaction. Moreover, Woods-Saxon parameterization is a very crude approximation to the nuclear mean-field. The true potential can be quite different and could include more complicated spin and tensor structures. The parameters of potential can change as a function of N and Z in a non-uniform manner. It is also found that the present calculation can only be used for certain values of *n* and *L* since the approximation to the centrifugal term $\frac{1}{\beta^{2}}$ is valid only for the lowest energy states [64].

The wave function is obtained by applying the value of α,ϑ, and z into equation (84). By using equations (85) and (89), (90) and (91), we get

(96) $$\alpha =\frac{1}{2}\left(\left(L^{\prime }-1-n\right)-\frac{\eta^{\prime }}{\left(L^{\prime }-1-n\right)}\right);\;\vartheta =\frac{1}{2}\left(\left(L^{\prime }-1-n\right)+\frac{\eta^{\prime }}{\left(L^{\prime }-1-n\right)}\right)$$

and

(97) $$a^{\prime }=-n;b^{\prime }=2L^{\prime }-1-n;c^{\prime }=\left(L^{\prime }-n\right)-\frac{\eta^{\prime }}{\left(L^{\prime }-1-n\right)}$$

By substituting equations (81), (96), and (97) into equation (84), we get the wave function in the case for rigid nucleus, γ=0 as

(98) $$\frac{R\left(\beta \right)}{\beta }={\left(\frac{1}{2}\right)}^{\left(L^{\prime }-1-n\right)}\left(\frac{\gamma^{\prime }}{\sinh\gamma^{\prime }\beta }\right){\left(1-\coth^{2}\gamma^{\prime }\beta \right)}^{\frac{\left(L^{\prime }-1-n\right)}{2}}{\left(\frac{1+\coth\gamma^{\prime }\beta }{1-\coth\gamma^{\prime }\beta }\right)}^{\frac{\eta^{\prime }}{2\left(L^{\prime }-1-n\right)}}F_{1}\left(a^{\prime };b^{\prime };c^{\prime };z\right)$$

The wave functions of Bohr Mottelson Hamiltonian equation for rigid γ=0 in the minimal length formalism for Woods-Saxon potential are obtained from equation (98) using the parameters constant values which are presented in Table 1 for n=0&n=1 that corresponds to the un-normalized ground state and first excited wave functions are given as

(99) $$\frac{R_{0}\left(\beta \right)}{\beta }={\left(\frac{1}{2}\right)}^{\left(L^{\prime }-1\right)}\left(\frac{\gamma^{\prime }}{\sinh\gamma^{\prime }\beta }\right){\left(1-\coth^{2}\gamma^{\prime }\beta \right)}^{\frac{\left(L^{\prime }-1\right)}{2}}{\left(\frac{1+\coth\gamma^{\prime }\beta }{1-\coth\gamma^{\prime }\beta }\right)}^{\frac{\eta^{\prime }}{2\left(L^{\prime }-1\right)}}$$

(100) $$\frac{R_{1}\left(\beta \right)}{\beta }={\left(\frac{1}{2}\right)}^{\left(L^{\prime }-2\right)}\left(\frac{\gamma^{\prime }}{\sinh\gamma^{\prime }\beta }\right){\left(1-\coth^{2}\gamma^{\prime }\beta \right)}^{\frac{\left(L^{\prime }-2\right)}{2}}{\left(\frac{1+\coth\gamma^{\prime }\beta }{1-\coth\gamma^{\prime }\beta }\right)}^{\frac{\eta^{\prime }}{2\left(L^{\prime }-1\right)}}\left(1-\frac{\left(2L^{\prime }-2\right)\left(1-\coth\gamma^{\prime }\beta \right)}{\left(L^{\prime }-1\right)-\frac{\eta^{\prime }}{\left(L^{\prime }-2\right)}}\right)$$

**Figure 3:**
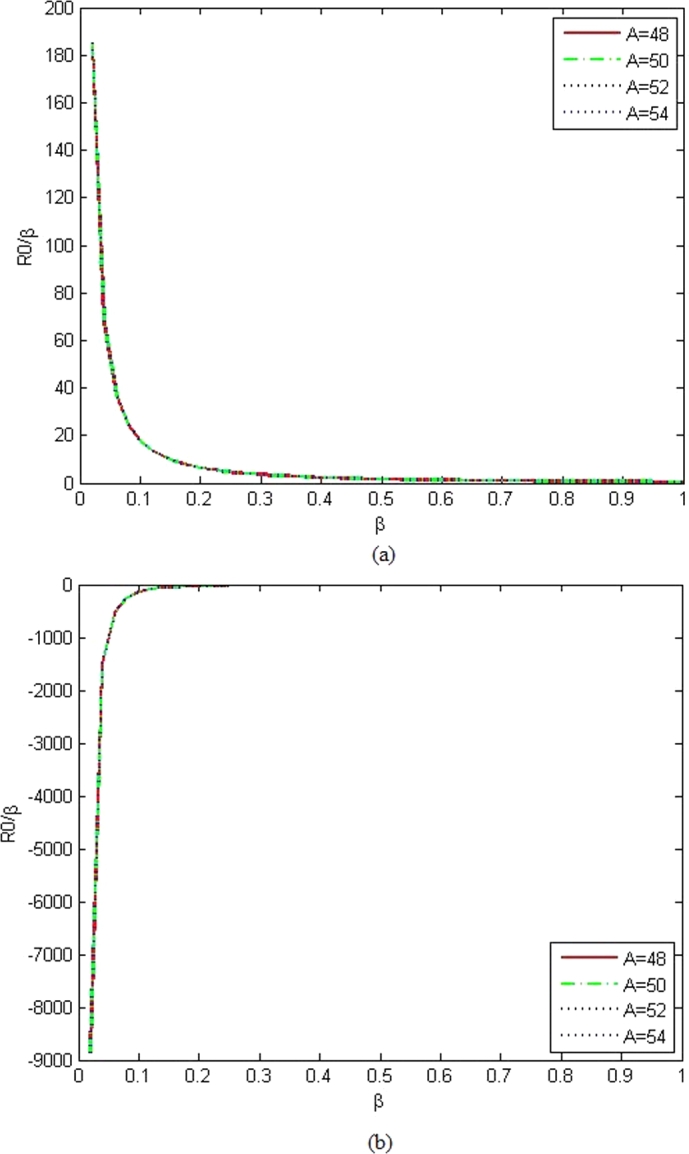
The graphs of the un-normalized ground state wave function (*R*_0_) as a function of *β* for different atoms with *α*_*ML*_ = 0.001 and (a) *L* = 1, (b) *L* = 3.

**Figure 4:**
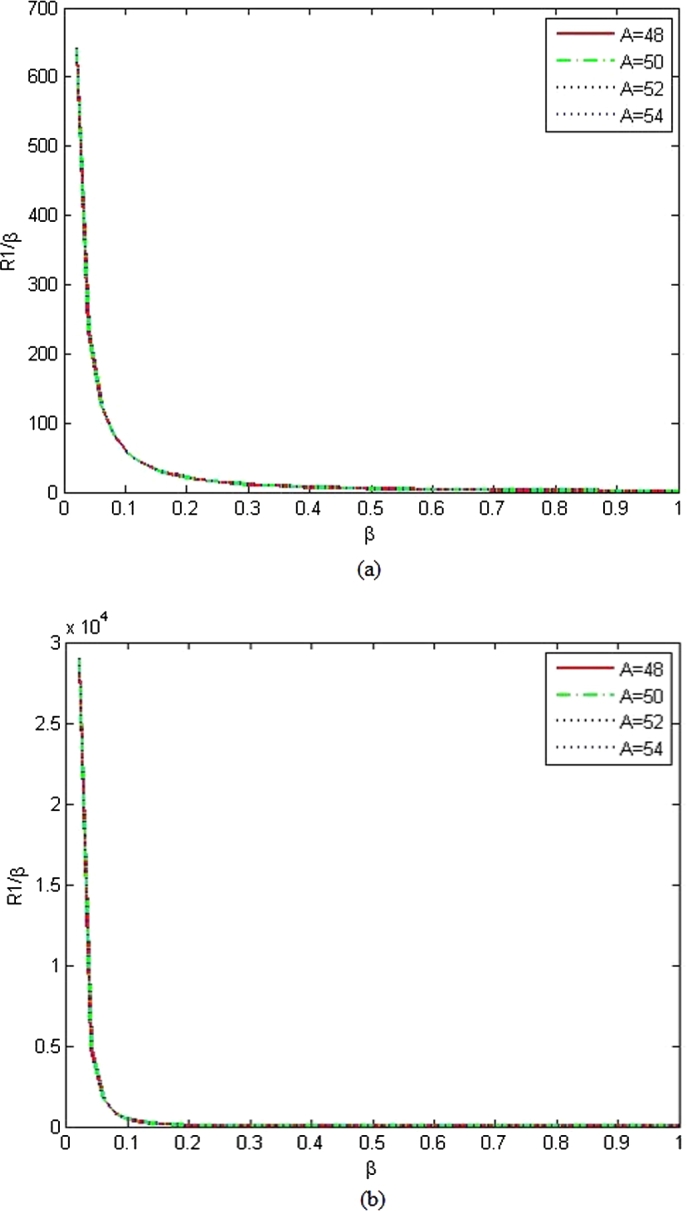
The graphs of the first excited states wave functions (*R*_1_) as a function of *β* for different atoms with *α*_*ML*_ = 0.001 and (a) *L* = 1, (b) *L* = 3.

The graphs of the un-normalized ground state and first excited state wave functions are shown in Figs. 3, 4 for various *L*.

### 6.2 Thermodynamic properties

The energy spectra expressed in equation (95) are used to determine the thermodynamical properties of the nucleus that are described using the Bohr Mottelson Hamiltonian equation with Woods-Saxon potential within the minimal length formalism.

The partition function which is defined as [46], [47], [48], [49]

(101) $$Z_{vib}\left(\beta^{\prime }\right)=\sum_{n=0}^{n_{max}}e^{-\beta^{\prime }E_{n}}=\sum_{n=0}^{n_{max}}f(\beta^{\prime },n_{max})$$

will be obtained using the energy spectra expressed in equation (95). However, since the energy eigenvalue of Bohr Mottelson Hamiltonian equation for Woods-Saxon potential in minimal length formalism is not able to be expressed explicitly only as functions of potential parameters, quantum numbers, and minimal length parameter, therefore the thermodynamics properties of a quantum system that is determined only for the condition when the minimal length parameter $\alpha_{\mathrm{ML}}$ is very small, thus the energy levels of Woods-Saxon potential in equation (95) reduce to

(102) $$E_{n}=-\frac{4\gamma^{\prime \;2}\hbar^{2}}{2B_{m}}{\left[\frac{\left(n+1-L^{\prime }\right)}{2}-\frac{\left(\frac{{\left(B_{m}\sigma V_{0}\right)}^{2}}{4\gamma^{\prime \;2}\hbar^{2}q}\right)}{\left(n+1-L^{\prime }\right)}\right]}^{2}$$

with $L^{\prime }=\frac{1}{2}+\sqrt{\frac{L\left(L+1\right)}{3}+\frac{1}{4}}$.

Equation (102) is simplified by setting

(103) $$\frac{4\gamma^{\prime \;2}\hbar^{2}}{2B_{m}}=\lambda ;\frac{B_{m}\sigma V_{0}}{2\gamma^{\prime \;2}\hbar^{2}q}=s;1-L^{\prime }=t$$

Applying equation (103) into equation (102) we obtain the following expression

(104) $$E_{n}=-\lambda {\left\{\frac{t+n}{2}-\frac{s}{2\left(t+n\right)}\right\}}^{2}$$

By inserting equation (104) into equation (101) we obtain the vibrational partition function given as

(105) $$Z_{vib}\left(\beta^{\prime }\right)=\sum_{n=0}^{n_{\max}}e^{-\beta^{\prime }\left(-\lambda {\left\{\frac{s}{2\left(t+n\right)}-\frac{t+n}{2}\right\}}^{2}\right)}=\sum_{n=0}^{n_{\max}}e^{\beta^{\prime }\lambda {\left\{\frac{s}{2\left(t+n\right)}-\frac{t+n}{2}\right\}}^{2}}$$

The vibrational energy levels expressed in equation (102) decrease with the increase of the value of quantum number *n*, therefore the closest to the maximum value of *n* allowed is determined from the condition that $\frac{dE_{n}}{dn}=0$,

(106) $$\frac{d}{dn}\left\{-\frac{4\gamma^{2}\hbar^{2}}{2B_{m}}\left[\frac{\left(n+1-L^{\prime }\right)}{2}-\frac{\left(\frac{{\left(B_{m}\sigma V_{0}\right)}^{2}}{4\gamma^{2}\hbar^{2}q}\right)}{\left(n+1-L^{\prime }\right)}\right]\right\}=0$$

that gives the maximum value of *n* as

(107) $$n_{mac}=L^{\prime }-1+\sqrt{\frac{B_{m}\sigma V_{0}}{2\gamma^{2}q}}$$

For this case, the partition function is recognized as the classical partition function, then the only lowest order approximation is being considered, and the Poisson summation formula for a finite summation with the upper bound $n_{\max}$ is expressed by [49]

(108) $$Z_{vib}\left(\beta^{\prime }\right)=\sum_{n=0}^{n_{max}}f\left(n\right)=\frac{1}{2}\left[f\left(0\right)-f\left(n_{max}+1\right)\right]+\int_{0}^{n_{max}+1}f\left(x\right)dx$$

By using equations (105) and (106), then equation (108) becomes

(109) $$Z_{vib}\left(\beta^{\prime }\right)=\sum_{n=0}^{n_{\max}}f\left(n\right)=\frac{1}{2}\left[e^{\beta^{\prime }\left(\lambda k_{1}^{2}\right)}-e^{\beta^{\prime }\left(\lambda k_{2}^{2}\right)}\right]+\int_{0}^{n_{\max}+1}e^{\beta^{\prime }\left(\lambda {\left(\frac{s}{2\left(t+x\right)}-\frac{\left(t+x\right)}{2}\right)}^{2}\right)}$$

with

(110) $$\frac{s}{t+1+n_{\max}}-\frac{t+1+n_{\max}}{2}=k_{2};f\left(0\right)=e^{\beta^{\prime }\left(\lambda k_{1}^{2}\right)};f\left(n_{\max}+1\right)=e^{\beta^{\prime }\left(\lambda k_{2}^{2}\right)}$$

and equation (109) provides the classical vibrational partition function, that is

(111) $$Z_{vib}(\beta^{\prime })=\frac{1}{2}\left\{\left[e^{\beta^{\prime }\left(\lambda k_{1}^{2}\right)}-e^{\beta^{\prime }\left(\lambda k_{2}^{2}\right)}\right]+\frac{\sqrt{\pi }}{\sqrt{\beta^{\prime }\lambda }}\left[\begin{array}{c} -\mathrm{erfi}\left(\sqrt{\beta^{\prime }\lambda }k_{1}\right)+\mathrm{erfi}\left(\sqrt{\beta^{\prime }\lambda }k_{2}\right)\\ -e^{-\beta^{\prime }\lambda s}\mathrm{erfi}\left(\sqrt{\beta^{\prime }\lambda \left(k_{1}^{2}+s\right)}\right)\\ +e^{-\beta^{\prime }\lambda s}\mathrm{erfi}\left(\sqrt{\beta^{\prime }\lambda \left(k_{2}^{2}+s\right)}\right) \end{array}\right]\right\}$$

**Figure 5:**
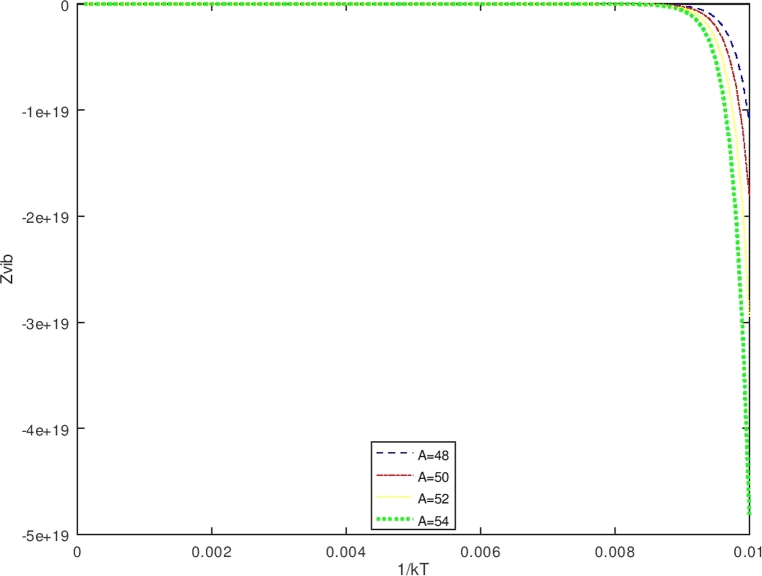
The graph of the vibrational partition function as a function of *β*.

Equation (111) is the vibrational partition function in the terms of *erfi* function, thus, using the thermodynamic formulas in equations (62), (63), (64) and (65), the thermodynamic functions can be determined from the partition function in equation (111) (see Fig. 5).

The vibrational mean energy *U* which is obtained from equations (62) and equation (111) results in

(112) $$U\left(\beta^{\prime }\right)=-\frac{\left\{\begin{array}{c} \left[\left(\lambda k_{1}^{2}\right)e^{\beta^{\prime }\left(\lambda k_{1}^{2}\right)}-\left(\lambda k_{2}^{2}\right)e^{\beta^{\prime }\left(\lambda k_{2}^{2}\right)}\right]-\frac{\sqrt{\pi }}{2\sqrt{\beta^{\prime \;3}\lambda }}\left[\begin{array}{c} -\mathrm{erfi}\left(\sqrt{\beta^{\prime }\lambda }k_{1}\right)+\mathrm{erfi}\left(\sqrt{\beta^{\prime }\lambda }k_{2}\right)\\ -e^{-\beta^{\prime }\lambda s}\mathrm{erfi}\left(\sqrt{\beta^{\prime }\lambda \left(k_{1}^{2}+s\right)}\right)\\ +e^{-\beta^{\prime }\lambda s}\mathrm{erfi}\left(\sqrt{\beta^{\prime }\lambda \left(k_{2}^{2}+s\right)}\right) \end{array}\right]\\ +\frac{1}{\beta^{\prime }}\left[\begin{array}{c} -e^{\beta^{\prime }\left(\lambda k_{1}^{2}\right)}k_{1}+e^{\beta^{\prime }\left(\lambda k_{2}^{2}\right)}k_{2}+\sqrt{\beta^{\prime }\pi \lambda }se^{-\beta^{\prime }\lambda s}\mathrm{erfi}\left(\sqrt{\beta^{\prime }\lambda \left(k_{1}^{2}+s\right)}\right)\\ -e^{\beta^{\prime }\left(\lambda k_{1}^{2}\right)}\sqrt{\left(k_{1}^{2}+s\right)}-\sqrt{\beta^{\prime }\pi \lambda }se^{-\beta^{\prime }\lambda s}\mathrm{erfi}\left(\sqrt{\beta^{\prime }\lambda \left(k_{2}^{2}+s\right)}\right)\\ +e^{\beta^{\prime }\left(\lambda k_{2}^{2}\right)}\sqrt{\left(k_{2}^{2}+s\right)} \end{array}\right] \end{array}\right\}}{\left\{\left[e^{\beta^{\prime }\left(\lambda k_{1}^{2}\right)}-e^{\beta^{\prime }\left(\lambda k_{2}^{2}\right)}\right]+\frac{\sqrt{\pi }}{\sqrt{\beta^{\prime }\lambda }}\left[\begin{array}{c} -\mathrm{erfi}\left(\sqrt{\beta^{\prime }\lambda }k_{1}\right)+\mathrm{erfi}\left(\sqrt{\beta^{\prime }\lambda }k_{2}\right)\\ -e^{-\beta^{\prime }\lambda s}\mathrm{erfi}\left(\sqrt{\beta^{\prime }\lambda \left(k_{1}^{2}+s\right)}\right)\\ +e^{-\beta^{\prime }\lambda s}\mathrm{erfi}\left(\sqrt{\beta^{\prime }\lambda \left(k_{2}^{2}+s\right)}\right) \end{array}\right]\right\}}$$

The vibrational specific heat (C) is obtained using equations (63) and (112), also by setting $U\left(\beta^{\prime }\right)=\frac{S_{2}}{S_{4}};S_{1}=\frac{d\left(\frac{1}{s_{4}}\right)}{d\beta^{\prime }};S_{3}=\frac{dS_{2}}{d\beta^{\prime }}$. One obtains the vibrational specific heat equation

(113) $$C\left(\beta^{\prime }\right)=k\beta^{\prime }\left(S_{1}\times S_{2}+\frac{S_{3}}{S_{4}}\right)$$

with

$$S_{1}=\frac{d\left(\frac{1}{s_{4}}\right)}{d\beta^{\prime }}=-{\left(S_{4}\right)}^{-2}\frac{dS_{4}}{d\beta^{\prime }}$$

(114) $$S_{1}=-\frac{\left\{\begin{array}{c} \left[\left(\lambda k_{1}^{2}\right)e^{\beta^{\prime }\left(\lambda k_{1}^{2}\right)}-\left(\lambda k_{2}^{2}\right)e^{\beta^{\prime }\left(\lambda k_{2}^{2}\right)}\right]-\frac{\sqrt{\pi }}{2\sqrt{\beta^{\prime \;3}\lambda }}\left[\begin{array}{c} -\mathrm{erfi}\left(\sqrt{\beta^{\prime }\lambda }k_{1}\right)+\mathrm{erfi}\left(\sqrt{\beta^{\prime }\lambda }k_{2}\right)\\ -e^{-\beta^{\prime }\lambda s}\mathrm{erfi}\left(\sqrt{\beta^{\prime }\lambda \left(k_{1}^{2}+s\right)}\right)\\ +e^{-\beta^{\prime }\lambda s}\mathrm{erfi}\left(\sqrt{\beta^{\prime }\lambda \left(k_{2}^{2}+s\right)}\right) \end{array}\right]\\ +\frac{1}{\beta^{\prime }}\left[\begin{array}{c} -e^{\beta^{\prime }\left(\lambda k_{1}^{2}\right)}k_{1}+e^{\beta^{\prime }\left(\lambda k_{2}^{2}\right)}k_{2}+\sqrt{\beta^{\prime }\pi \lambda }se^{-\beta^{\prime }\lambda s}\mathrm{erfi}\left(\sqrt{\beta^{\prime }\lambda \left(k_{1}^{2}+s\right)}\right)\\ -e^{\beta^{\prime }\left(\lambda k_{1}^{2}\right)}\sqrt{\left(k_{1}^{2}+s\right)}-\sqrt{\beta^{\prime }\pi \lambda }se^{-\beta^{\prime }\lambda s}\mathrm{erfi}\left(\sqrt{\beta^{\prime }\lambda \left(k_{2}^{2}+s\right)}\right)\\ +e^{\beta^{\prime }\left(\lambda k_{2}^{2}\right)}\sqrt{\left(k_{2}^{2}+s\right)} \end{array}\right] \end{array}\right\}}{{\left\{\left[e^{\beta^{\prime }\left(\lambda k_{1}^{2}\right)}-e^{\beta^{\prime }\left(\lambda k_{2}^{2}\right)}\right]+\frac{\sqrt{\pi }}{\sqrt{\beta^{\prime }\lambda }}\left[\begin{array}{c} -\mathrm{erfi}\left(\sqrt{\beta^{\prime }\lambda }k_{1}\right)+\mathrm{erfi}\left(\sqrt{\beta^{\prime }\lambda }k_{2}\right)\\ -e^{-\beta^{\prime }\lambda s}\mathrm{erfi}\left(\sqrt{\beta^{\prime }\lambda \left(k_{1}^{2}+s\right)}\right)\\ +e^{-\beta^{\prime }\lambda s}\mathrm{erfi}\left(\sqrt{\beta^{\prime }\lambda \left(k_{2}^{2}+s\right)}\right) \end{array}\right]\right\}}^{2}}$$

(115) $$S_{2}=\left\{\begin{array}{c} \left[\left(\lambda k_{1}^{2}\right)e^{\beta^{\prime }\left(\lambda k_{1}^{2}\right)}-\left(\lambda k_{2}^{2}\right)e^{\beta^{\prime }\left(\lambda k_{2}^{2}\right)}\right]-\frac{\sqrt{\pi }}{2\sqrt{\beta^{\prime \;3}\lambda }}\left[\begin{array}{c} -\mathrm{erfi}\left(\sqrt{\beta^{\prime }\lambda }k_{1}\right)+\mathrm{erfi}\left(\sqrt{\beta^{\prime }\lambda }k_{2}\right)\\ -e^{-\beta^{\prime }\lambda s}\mathrm{erfi}\left(\sqrt{\beta^{\prime }\lambda \left(k_{1}^{2}+s\right)}\right)\\ +e^{-\beta^{\prime }\lambda s}\mathrm{erfi}\left(\sqrt{\beta^{\prime }\lambda \left(k_{2}^{2}+s\right)}\right) \end{array}\right]\\ +\frac{1}{\beta^{\prime }}\left[\begin{array}{c} -e^{\beta^{\prime }\left(\lambda k_{1}^{2}\right)}k_{1}+e^{\beta^{\prime }\left(\lambda k_{2}^{2}\right)}k_{2}+\sqrt{\beta^{\prime }\pi \lambda }se^{-\beta^{\prime }\lambda s}\mathrm{erfi}\left(\sqrt{\beta^{\prime }\lambda \left(k_{1}^{2}+s\right)}\right)\\ -e^{\beta^{\prime }\left(\lambda k_{1}^{2}\right)}\sqrt{\left(k_{1}^{2}+s\right)}-\sqrt{\beta^{\prime }\pi \lambda }se^{-\beta^{\prime }\lambda s}\mathrm{erfi}\left(\sqrt{\beta^{\prime }\lambda \left(k_{2}^{2}+s\right)}\right)\\ +e^{\beta^{\prime }\left(\lambda k_{2}^{2}\right)}\sqrt{\left(k_{2}^{2}+s\right)} \end{array}\right] \end{array}\right\}$$

(116) $$S_{3}=\frac{dS_{2}}{d\beta^{\prime }}$$

(117) $$S_{3}=\left\{\begin{array}{c} \left[\left(\lambda k_{1}^{2}\right)e^{\beta^{\prime }\left(\lambda k_{1}^{2}\right)}-\left(\lambda k_{2}^{2}\right)e^{\beta^{\prime }\left(\lambda k_{2}^{2}\right)}\right]+\frac{3\sqrt{\pi }}{4\sqrt{\beta^{\prime \;5}\lambda }}\left[\begin{array}{c} -\mathrm{erfi}\left(\sqrt{\beta^{\prime }\lambda }k_{1}\right)+\mathrm{erfi}\left(\sqrt{\beta^{\prime }\lambda }k_{2}\right)\\ -e^{-\beta^{\prime }\lambda s}\mathrm{erfi}\left(\sqrt{\beta^{\prime }\lambda \left(k_{1}^{2}+s\right)}\right)\\ +e^{-\beta^{\prime }\lambda s}\mathrm{erfi}\left(\sqrt{\beta^{\prime }\lambda \left(k_{2}^{2}+s\right)}\right) \end{array}\right]\\ -\frac{3}{2\beta^{\prime }}\left[\begin{array}{c} -e^{\beta^{\prime }\left(\lambda k_{1}^{2}\right)}k_{1}+e^{\beta^{\prime }\left(\lambda k_{2}^{2}\right)}k_{2}+\sqrt{\beta^{\prime }\pi \lambda }se^{-\beta^{\prime }\lambda s}\mathrm{erfi}\left(\sqrt{\beta^{\prime }\lambda \left(k_{1}^{2}+s\right)}\right)\\ -e^{\beta^{\prime }\left(\lambda k_{1}^{2}\right)}\sqrt{\left(k_{1}^{2}+s\right)}-\sqrt{\beta^{\prime }\pi \lambda }se^{-\beta^{\prime }\lambda s}\mathrm{erfi}\left(\sqrt{\beta^{\prime }\lambda \left(k_{2}^{2}+s\right)}\right)\\ +e^{\beta^{\prime }\left(\lambda k_{2}^{2}\right)}\sqrt{\left(k_{2}^{2}+s\right)} \end{array}\right]\\ +\frac{1}{\beta^{\prime }}\left[\begin{array}{c} -\lambda k_{1}^{2}e^{\beta^{\prime }\left(\lambda k_{1}^{2}\right)}k_{1}+\lambda k_{2}^{2}e^{\beta^{\prime }\left(\lambda k_{2}^{2}\right)}k_{2}+\frac{1}{2}\sqrt{\frac{\pi \lambda }{\beta^{\prime }}}se^{-\beta^{\prime }\lambda s}\left(\sqrt{\beta^{\prime }\lambda \left(k_{1}^{2}+s\right)}\right)\\ -\left(\begin{array}{c} \left(\lambda s\right)\sqrt{\beta^{\prime }\pi \lambda }se^{-\beta^{\prime }\lambda s}\\ \mathrm{erfi}\left(\sqrt{\beta^{\prime }\lambda \left(k_{1}^{2}+s\right)}\right) \end{array}\right)+\sqrt{\beta^{\prime }\pi \lambda }se^{-\beta^{\prime }\lambda s}\frac{2}{\sqrt{\pi }}e^{\beta^{\prime }\lambda \left(k_{1}^{2}+s\right)}\frac{1}{2}\sqrt{\frac{\lambda }{\beta^{\prime }}\left(k_{1}^{2}+s\right)}\\ -\lambda k_{1}^{2}e^{\beta^{\prime }\left(\lambda k_{1}^{2}\right)}\left(\sqrt{\left(k_{1}^{2}+s\right)}\right)-\frac{1}{2}\sqrt{\frac{\pi \lambda }{\beta^{\prime }}}se^{-\beta^{\prime }\lambda s}\left(\sqrt{\beta^{\prime }\lambda \left(k_{2}^{2}+s\right)}\right)+\left(\begin{array}{c} \left(\lambda s\right)\sqrt{\beta^{\prime }\pi \lambda }se^{-\beta^{\prime }\lambda s}\\ \mathrm{erfi}\left(\sqrt{\beta^{\prime }\lambda \left(k_{2}^{2}+s\right)}\right) \end{array}\right)\\ -\sqrt{\beta^{\prime }\pi \lambda }se^{-\beta^{\prime }\lambda s}\frac{2}{\sqrt{\pi }}e^{\beta^{\prime }\lambda \left(k_{2}^{2}+s\right)}\frac{1}{2}\sqrt{\frac{\lambda }{\beta^{\prime }}\left(k_{2}^{2}+s\right)}+\lambda k_{2}^{2}e^{\beta^{\prime }\left(\lambda k_{2}^{2}\right)}\left(\sqrt{\left(k_{2}^{2}+s\right)}\right) \end{array}\right] \end{array}\right\}$$

(118) $$S_{4}=\left\{\left[e^{\beta^{\prime }\left(\lambda k_{1}^{2}\right)}-e^{\beta^{\prime }\left(\lambda k_{2}^{2}\right)}\right]+\frac{\sqrt{\pi }}{\sqrt{\beta^{\prime }\lambda }}\left[\begin{array}{c} -\mathrm{erfi}\left(\sqrt{\beta^{\prime }\lambda }k_{1}\right)+\mathrm{erfi}\left(\sqrt{\beta^{\prime }\lambda }k_{2}\right)\\ -e^{-\beta^{\prime }\lambda s}\mathrm{erfi}\left(\sqrt{\beta^{\prime }\lambda \left(k_{1}^{2}+s\right)}\right)\\ +e^{-\beta^{\prime }\lambda s}\mathrm{erfi}\left(\sqrt{\beta^{\prime }\lambda \left(k_{2}^{2}+s\right)}\right) \end{array}\right]\right\}$$

Furthermore, the vibrational free energy (F) which is obtained using equations (64) and (111) is given as

(119) $$F\left(\beta^{\prime }\right)=-\frac{1}{\beta^{\prime }}\ln\frac{1}{2}\left\{\left[e^{\beta^{\prime }\left(\lambda k_{1}^{2}\right)}-e^{\beta^{\prime }\left(\lambda k_{2}^{2}\right)}\right]+\frac{\sqrt{\pi }}{\sqrt{\beta^{\prime }\lambda }}\left[\begin{array}{c} -\mathrm{erfi}\left(\sqrt{\beta^{\prime }\lambda }k_{1}\right)+\mathrm{erfi}\left(\sqrt{\beta^{\prime }\lambda }k_{2}\right)\\ -e^{-\beta^{\prime }\lambda s}\mathrm{erfi}\left(\sqrt{\beta^{\prime }\lambda \left(k_{1}^{2}+s\right)}\right)\\ +e^{-\beta^{\prime }\lambda s}\mathrm{erfi}\left(\sqrt{\beta^{\prime }\lambda \left(k_{2}^{2}+s\right)}\right) \end{array}\right]\right\}$$

While the vibrational entropy S is determined using equations (65) and (111), it has the form as

$$S\left(\beta^{\prime }\right)=k\ln Z_{vib}\left(\beta^{\prime }\right)+kT\frac{\partial }{\partial T}\ln Z_{vib}\left(\beta^{\prime }\right)$$

(120) $$S\left(\beta^{\prime }\right)=k\ln\frac{1}{2}\left\{\left[e^{\beta^{\prime }\left(\lambda k_{1}^{2}\right)}-e^{\beta^{\prime }\left(\lambda k_{2}^{2}\right)}\right]+\frac{\sqrt{\pi }}{\sqrt{\beta^{\prime }\lambda }}\left[\begin{array}{c} -\mathrm{erfi}\left(\sqrt{\beta^{\prime }\lambda }k_{1}\right)+\mathrm{erfi}\left(\sqrt{\beta^{\prime }\lambda }k_{2}\right)\\ -e^{-\beta^{\prime }\lambda s}\mathrm{erfi}\left(\sqrt{\beta^{\prime }\lambda \left(k_{1}^{2}+s\right)}\right)\\ +e^{-\beta^{\prime }\lambda s}\mathrm{erfi}\left(\sqrt{\beta^{\prime }\lambda \left(k_{2}^{2}+s\right)}\right) \end{array}\right]\right\}+k\beta^{\prime }\left[U\left(\beta^{\prime }\right)\right]$$

with $U\left(\beta^{\prime }\right)$ is expressed in equation (112).

## 7 Conclusion

**Figure 6:**
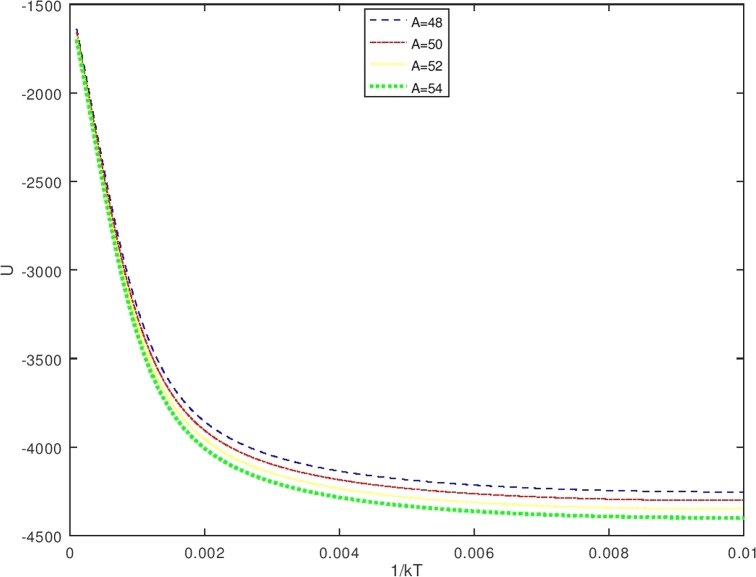
The graph of the vibrational mean energy as a function of *β*.

**Figure 7:**
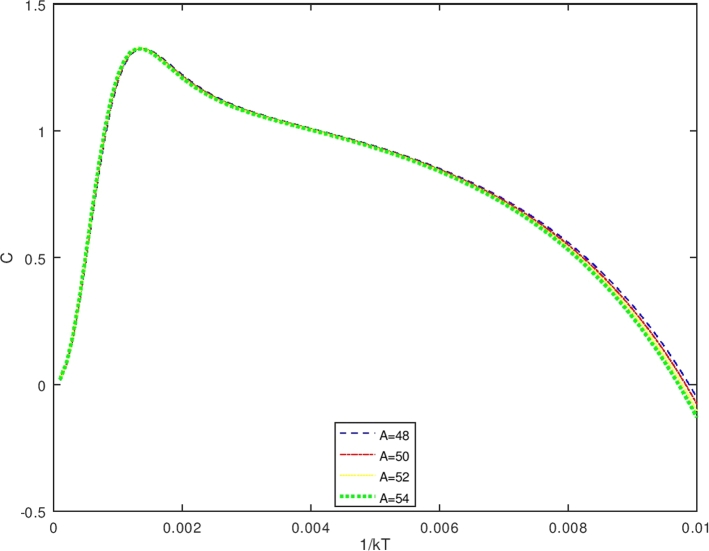
The graph of the vibrational specific heat as a function of *β*.

**Figure 8:**
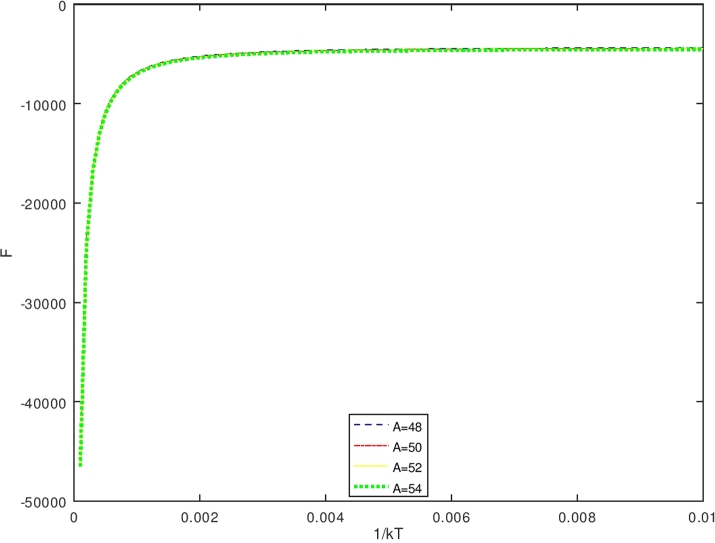
The graph of the vibrational free energy as a function of *β*.

**Figure 9:**
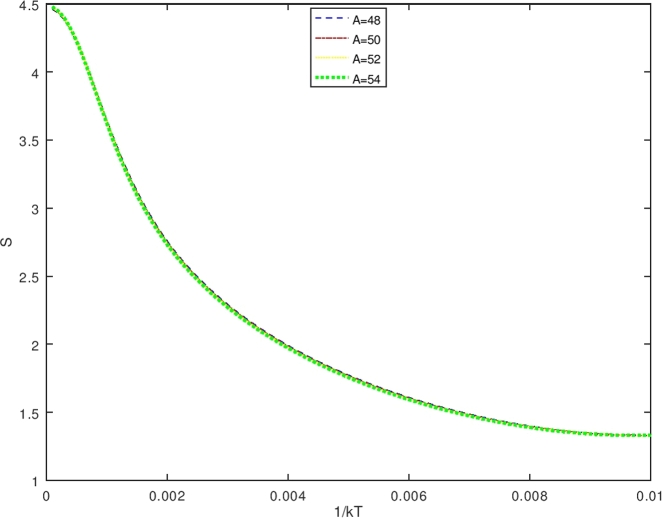
The graph of the vibrational entropy as a function of *β*.

The Bohr Mottelson Hamiltonian in the *β* collective shape variable for the Woods-Saxon potential was investigated in the effect of the minimal length formalism for γ=0 in the rigid deformed nucleus with the X(3) model. The equation was solved approximately by introducing a new wave function. Then, it was reduced to the Schrodinger-like equation with cotangent hyperbolic potential by using the q deformed hyperbolic potential concept such that the rigid deformed nucleus of the Bohr Mottelson equation in the minimal length formalism for Woods-Saxon potential. The hypergeometric method was used to obtain the energy spectra equation and the unnormalized wave function of the system shown in equations (95) and respectively. The calculated energy spectra with the variation of radial quantum number (*n*), the angular quantum number (*L*), and minimal length parameter values $\alpha_{\mathrm{ML}}$ are shown in Tables 2, 3, 4, 5, 6, 7 and 8 and the corresponding graphs of the calculated energy spectra with $\alpha_{\mathrm{ML}}=0$ are shown in Figs. 1, 2. Figs. 1, 2 show that the graph of energy without the effect of minimal length, the maximum values of the energy of each value of n depend on the values of radial and orbital quantum numbers n and L. The greater the value of n, the maximum value of the energy is shifted toward the greater value of L. The energy spectra for four atoms with the presence of minimal length are shown in Tables 7, 8. Tables 7, 8 show that the minimal length parameter has the most effect on the energy spectra. The larger is the values of the minimal length the higher is the energy spectra of the atoms. Also, from Tables 2, 3, 4, 5 and 6 and Tables 7, 8 could be concluded that the values of the energy spectra for the similar value of n and L but different values of minimal length are the same. The thermodynamic properties are calculated analytically, shown in equations (112), (113) and (119), (120). Then, it is plotted into the graph shown in Figs. 6, 7, 8 and 9.

## Declarations

### Author contribution statement

A. Suparmi: Conceived and designed the experiments; Wrote the paper.

L.K. Permatahati: Performed the experiments; Analyzed and interpreted the data; Wrote the paper.

S. Faniandari: Analyzed and interpreted the data; Wrote the paper.

Y. Iriani, A. Marzuki: Contributed reagents, materials, analysis tools or data.

### Funding statement

A. Suparmi was supported by 10.13039/501100007690Universitas Sebelas Maret (260/UN27.22/HK.07.00/2021).

### Data availability statement

Data included in article/supplementary material/referenced in article.

### Declaration of interests statement

The authors declare no conflict of interest.

### Additional information

No additional information is available for this paper.

## Appendix A

The Heisenberg Uncertainty Principle defined as

(1) $$\left[\overset{ˆ}{x},\overset{ˆ}{p}\right]\geq ih$$

The GUP equation shows the modified canonical commutation relation as given by

(2) $$\left[X,P\right]=i\hbar \left(1+\alpha_{\mathrm{ML}}p^{2}\right)$$

The deformed uncertainty of canonical commutation relation leads to the deformed uncertainty relation expressed as

(3) $$\Delta X\Delta P=\frac{\hbar }{2}\left(1+\alpha_{\mathrm{ML}}{\left(\Delta p\right)}^{2}\right)$$

From equation (3), we have the minimal length

(4) $${\left(\Delta X\right)}_{\mathrm{ML}}=\hbar \sqrt{\alpha_{\mathrm{ML}}}$$

The position and the momentum operators commute each other, that is

(5) $${\overset{ˆ}{X}}_{i}={\overset{ˆ}{x}}_{i}$$

(6) $${\overset{ˆ}{P}}_{i}=\left(1+\alpha_{\mathrm{ML}}{\overset{ˆ}{p}}^{2}\right){\overset{ˆ}{p}}_{i}$$

The squared of the momentum operator at low energy is expressed by [35]

(7) $${\overset{ˆ}{p}}^{2}=-\hbar^{2}\Delta$$

at high energy, the squared of the momentum operator is obtained by substituting Equation (7) into equation (6), we have the form

(8) $${\overset{ˆ}{P}}^{2}=-\hbar^{2}\left(1-2\alpha_{\mathrm{ML}}\hbar^{2}\Delta \right)\Delta$$

The Laplacian operator for a nucleus has the form

(9) $$\Delta =\frac{1}{\sqrt{g}}\sum_{i,j}\frac{\partial }{\partial q_{i}}\sqrt{g}g_{ij}^{-1}\frac{\partial }{\partial q_{j}}$$

The collective geometrical model of the nucleus in curvilinear coordinates [2], [15] is introduced as

(10) $$ds^{2}=\sum_{i,j}g_{ij}dx_{i}dx_{j}$$

the metric tensor is constructed as

(11) $$g_{ij}=\left(\begin{array}{ccc} 3\beta^{2}\sin^{2}\theta \; & 0\; & 0\\ 0\; & 3\beta^{2}\; & 0\\ 0\; & 0\; & 1 \end{array}\right)$$

the determinant of the matrix results in

(12) $$g=9\beta^{2}\sin^{2}\theta$$

and the inverse of the matrix $g_{ij}$ is

(13) $$g_{ij}^{-1}=\left(\begin{array}{ccc} \frac{1}{3\beta^{2}\sin^{2}\theta }\; & 0\; & 0\\ 0\; & \frac{1}{3\beta^{2}}\; & 0\\ 0\; & 0\; & 1 \end{array}\right)$$

Matrix diagonal of equation (13) is

$$g_{11}^{-1}=\frac{1}{3\beta^{2}\sin^{2}\theta }\;g_{22}^{-1}=\frac{1}{3\beta^{2}}\;g_{33}^{-1}=1$$

By inserting equation (12), (13) into equation (9), the Laplacian operator has the following form

$$\Delta =\frac{1}{\sqrt{g}}\left[\frac{\partial }{\partial q_{1}}\left(\sqrt{g}g_{11}^{-1}\frac{\partial }{\partial q_{1}}\right)+\frac{\partial }{\partial q_{2}}\left(\sqrt{g}g_{22}^{-1}\frac{\partial }{\partial q_{2}}\right)+\frac{\partial }{\partial q_{3}}\left(\sqrt{g}g_{33}^{-1}\frac{\partial }{\partial q_{3}}\right)\right]$$

$$\Delta =\left[\frac{1}{\sqrt{9\beta^{2}\sin^{2}\theta }}\left[\begin{array}{c} \frac{\partial }{\partial \phi }\left(\sqrt{9\beta^{2}\sin^{2}\theta }\frac{1}{3\beta^{2}\sin^{2}\theta }\frac{\partial }{\partial \phi }\right)\\ +\frac{\partial }{\partial \theta }\left(\sqrt{9\beta^{2}\sin^{2}\theta }\frac{1}{3\beta^{2}}\frac{\partial }{\partial \theta }\right)+\frac{\partial }{\partial \beta }\left(\sqrt{9\beta^{2}\sin^{2}\theta }\frac{\partial }{\partial \beta }\right) \end{array}\right]\right]$$

the Laplacian operator becomes

(14) $$\Delta =\frac{1}{\beta^{2}}\frac{\partial }{\partial \beta }\left(\beta^{2}\frac{\partial }{\partial \beta }\right)+\frac{1}{3\beta^{2}}\left[\frac{1}{\sin^{2}\theta }\frac{\partial^{2}}{\partial \varphi^{2}}+\frac{1}{\sin\theta }\frac{\partial }{\partial \theta }\left(\sin\theta \frac{\partial }{\partial \theta }\right)\right]$$

with $\left[\frac{1}{\sin^{2}\theta }\frac{\partial^{2}}{\partial \varphi^{2}}+\frac{1}{\sin\theta }\frac{\partial }{\partial \theta }\left(\sin\theta \frac{\partial }{\partial \theta }\right)\right]=\Delta_{\Omega }$ is Euler angles part of Bohr Mottelson Hamiltonian equation.

The Hamiltonian equation is defined as [7]

(15) $$H=T+V\left(\beta \right)=\frac{p^{2}}{2B_{m}}+V\left(\beta \right)$$

Equation (8) is inserted into equation (15), so we get

$$H=\frac{-\hbar^{2}\left(1-2\alpha_{\mathrm{ML}}\hbar^{2}\Delta \right)\Delta }{2B_{m}}+V\left(\beta \right)$$

(16) $$H=-\frac{\hbar^{2}}{2B_{m}}\Delta +\frac{\alpha_{\mathrm{ML}}\hbar^{4}}{B_{m}}\Delta^{2}+V\left(\beta ,\theta ,\phi \right)$$

or it can be rewritten as

(17) $$\left[-\frac{\hbar^{2}}{2B_{m}}\Delta +\frac{\alpha_{\mathrm{ML}}\hbar^{4}}{B_{m}}\Delta^{2}+V\left(\beta ,\theta ,\phi \right)-E\right]\psi \left(\beta ,\theta ,\phi \right)=0$$

To solve equation (17), the new wave function is introduced as follows

(18) $$\Psi \left(\beta ,\theta ,\phi \right)=\left(1+2\alpha_{\mathrm{ML}}\hbar^{2}\Delta \right)\chi \left(\beta ,\theta ,\phi \right)$$

By inserting equation (18) into equation (17), we have

(19) $$\left\{\frac{-\hbar^{2}\Delta }{2B_{m}}+\frac{\alpha_{\mathrm{ML}}\hbar^{4}\Delta^{2}}{B_{m}}+V\left(\beta \right)-E\right\}\left(1+2\alpha_{\mathrm{ML}}\hbar^{2}\Delta \right)\chi \left(\beta ,\theta ,\phi \right)=0$$

From equation (19) we have

(19a) $$\left\{\frac{-\hbar^{2}\Delta }{2B_{m}}-\frac{\alpha_{\mathrm{ML}}\hbar^{4}\Delta^{2}}{B_{m}}+\frac{\alpha_{\mathrm{ML}}\hbar^{4}\Delta^{2}}{B_{m}}+\frac{2\alpha_{\mathrm{ML}}^{2}\hbar^{6}\Delta^{3}}{B_{m}}+\left(V\left(\beta \right)-E\right)\left(1+2\alpha_{\mathrm{ML}}\hbar^{2}\Delta \right)\right\}\chi \left(\beta ,\theta ,\phi \right)=0$$

Since the $\alpha_{\mathrm{ML}}^{2}\approx 0$ then equation (19a) reduces to

(19b) $$\left\{\frac{-\hbar^{2}\Delta }{2B_{m}}+\left(V\left(\beta \right)-E\right)\left(1+2\alpha_{\mathrm{ML}}\hbar^{2}\Delta \right)\right\}\chi \left(\beta ,\theta ,\phi \right)=0$$

Then, the equation (19b) is multiplied by $-\frac{2B_{m}}{\hbar^{2}}$ we obtain

$$\left[\Delta \chi \left(\beta ,\theta ,\phi \right)-4B_{m}\alpha_{\mathrm{ML}}\left(V\left(\beta ,\theta ,\phi \right)-E\right)\Delta \chi \left(\beta ,\theta ,\phi \right)-\frac{2B_{m}}{\hbar^{2}}\left(V\left(\beta ,\theta ,\phi \right)-E\right)\chi \left(\beta ,\theta ,\phi \right)\right]=0$$

this equation reduces to

$$\left[\Delta \chi \left(\beta ,\theta ,\phi \right)-4B_{m}\alpha_{\mathrm{ML}}\left(V\left(\beta ,\theta ,\phi \right)-E\right)\Delta \chi \left(\beta ,\theta ,\phi \right)-\frac{2B_{m}}{\hbar^{2}}\left(V\left(\beta ,\theta ,\phi \right)-E\right)\chi \left(\beta ,\theta ,\phi \right)\right]=0$$

(20) $$\left\{\left(1+4\alpha_{\mathrm{ML}}B_{m}\left(E-V\left(\beta \right)\right)\right)\Delta +\frac{2B_{m}}{\hbar^{2}}\left(E-V\left(\beta \right)\right)\right\}\chi \left(\beta ,\theta ,\phi \right)=0$$

That reduces to

$$\Delta \chi \left(\beta ,\theta ,\phi \right)=\left[\frac{\frac{2B_{m}}{\hbar^{2}}\left(V-E\right)}{\left(1-4B_{m}\alpha_{\mathrm{ML}}\left(V-E\right)\right)}\right]\chi \left(\beta ,\theta ,\phi \right)\Delta \chi \left(\beta ,\theta ,\phi \right)=\left[\frac{-\frac{2B_{m}}{\hbar^{2}}E\left(1-\frac{V}{E}\right)}{\left(1+4B_{m}\alpha_{\mathrm{ML}}E\left(1-\frac{4B_{m}\alpha_{\mathrm{ML}}V}{1+4B_{m}\alpha_{\mathrm{ML}}E}\right)\right)}\right]\chi \left(\beta ,\theta ,\phi \right)$$

(20) $$\left[\Delta \chi \left(\beta ,\theta ,\phi \right)\left(1-4B_{m}\alpha_{\mathrm{ML}}\left(V-E\right)\right)-\frac{2B_{m}}{\hbar^{2}}\left(V-E\right)\chi \left(\beta ,\theta ,\phi \right)\right]=0$$

$$\Delta \chi \left(\beta ,\theta ,\phi \right)=\left[\frac{\frac{2B_{m}}{\hbar^{2}}\left(V-E\right)}{\left(1-4B_{m}\alpha_{\mathrm{ML}}\left(V-E\right)\right)}\right]\chi \left(\beta ,\theta ,\phi \right)\Delta \chi \left(\beta ,\theta ,\phi \right)=\left[\frac{-\frac{2B_{m}}{\hbar^{2}}E\left(1-\frac{V}{E}\right)}{\left(1+4B_{m}\alpha_{\mathrm{ML}}E\left(1-\frac{4B_{m}\alpha_{\mathrm{ML}}V}{1+4B_{m}\alpha_{\mathrm{ML}}E}\right)\right)}\right]\chi \left(\beta ,\theta ,\phi \right)$$

Equation (20) take the form

(21) $$\Delta \chi \left(\beta ,\theta ,\phi \right)+\left[\frac{\frac{2B_{m}}{\hbar^{2}}E\left(1-\frac{V}{E}\right)}{\left(1+4B_{m}\alpha_{\mathrm{ML}}E\left(1-\frac{4B_{m}\alpha_{\mathrm{ML}}V}{1+4B_{m}\alpha_{\mathrm{ML}}E}\right)\right)}\right]\chi \left(\beta ,\theta ,\phi \right)=0$$

to obtain the solution of equation (21) simply, we should set new parameters as

(22) $$\frac{2B_{m}}{\hbar^{2}}E=\omega ;1+4B_{m}\alpha_{\mathrm{ML}}E=\mu ;\frac{4B_{m}\alpha_{\mathrm{ML}}}{1+4B_{m}\alpha_{\mathrm{ML}}E}=-\tau ;E=\frac{1}{\varepsilon }$$

equation (21) is found to be

(23) $$\Delta \chi \left(\beta ,\theta ,\phi \right)+\frac{\omega }{\mu }\left[\frac{\left(1-\varepsilon V\right)}{\left(1+\tau V\right)}\right]\chi \left(\beta ,\theta ,\phi \right)=0$$

and simply reduces to

(24) $$\Delta \chi \left(\beta ,\theta ,\phi \right)+\frac{\omega }{\mu }\left[1+\frac{\left(-\tau -\varepsilon \right)V}{\left(1+\tau V\right)}\right]\chi \left(\beta ,\theta ,\phi \right)=0$$

By substituting equation (14) into equation (24), the Bohr Mottelson Hamiltonian equation for the certain energy potential V(β) appears in the following term

(25) $$\left\{\frac{\partial^{2}}{\partial \beta^{2}}+\frac{2}{\beta }\frac{\partial }{\partial \beta }+\frac{1}{3\beta^{2}}\left[\frac{1}{\sin^{2}\theta }\frac{\partial^{2}}{\partial \varphi^{2}}+\frac{1}{\sin\theta }\frac{\partial }{\partial \theta }\left(\sin\theta \frac{\partial }{\partial \theta }\right)\right]\right\}\chi \left(\beta ,\theta ,\phi \right)+\frac{\omega }{\mu }\left[1+\frac{\left(-\tau -\varepsilon \right)V}{\left(1+\tau V\right)}\right]\chi \left(\beta ,\theta ,\phi \right)=0$$

The latter equation can be solved using the following wave function

(26) $$\chi \left(\beta ,\theta ,\phi \right)=\frac{R\left(\beta \right)}{\beta }P(\theta )\Phi (\phi )$$

And applied into equation (25)

$$\left\{\frac{3\beta^{2}}{R\beta^{2}}\frac{\partial }{\partial \beta }\left(\beta^{2}\frac{\partial }{\partial \beta }\left(\frac{R}{\beta }\right)\right)+\left[\begin{array}{c} \frac{1}{P\sin\;\theta }\frac{\partial }{\partial \theta }\left(\sin\;\theta \frac{\partial P}{\partial \theta }\right)\\ +\frac{1}{\Phi \sin^{2}\theta }\frac{\partial^{2}\Phi }{\partial \phi^{2}} \end{array}\right]\right\}+\frac{\omega }{\mu }\left[1+\frac{\left(-\tau -\varepsilon \right)V}{\left(1+\tau V\right)}\right]3\beta^{2}=0$$

we obtain

(27) $$\left[\frac{1}{\Phi \sin^{2}\theta }\frac{\partial^{2}\Phi }{\partial \varphi^{2}}+\frac{1}{P\sin\theta }\frac{\partial }{\partial \theta }\left(\sin\theta \frac{\partial P}{\partial \theta }\right)\right]+3\beta^{2}\left[\frac{\partial^{2}R}{R\partial \beta^{2}}-\frac{\omega }{\mu }\frac{\left(\tau +\varepsilon \right)V}{\left(1+\tau V\right)}+\frac{\omega }{\mu }\right]=0$$

and we get the Bohr Mottelson Hamiltonian equation within the minimal length formalism for the *β* function as

(28) $$\frac{\partial^{2}R}{\partial \beta^{2}}-\frac{\omega }{\mu }\frac{\left(\tau +\varepsilon \right)V}{\left(1+\tau V\right)}R+\frac{\omega }{\mu }R-\frac{\delta }{3\beta^{2}}R=0$$

and for the angular part of the Bohr Mottelson Hamiltonian equation

(29) $$\left[\frac{1}{\Phi \sin^{2}\theta }\frac{\partial^{2}\Phi }{\partial \varphi^{2}}+\frac{1}{P\sin\theta }\frac{\partial }{\partial \theta }\left(\sin\theta \frac{\partial P}{\partial \theta }\right)\right]=-\delta$$

Equation (29) allows us to separate the angular equation into two ordinary differential equations, one being a function of (*θ*) and the other a function of (*ϕ*). This means we separate into

$$-\frac{1}{P}\frac{1}{\sin\theta }\frac{\partial }{\partial \theta }\left(\sin\theta \frac{\partial P}{\partial \theta }\right)=\frac{1}{\Phi }\frac{1}{\sin^{2}\theta }\frac{\partial^{2}\Phi }{\partial \varphi^{2}}=\delta$$

If we multiply each term by $-\sin^{2}\theta$, with δ=L(L+1)

$$\frac{\sin\theta }{P}\frac{\partial }{\partial \theta }\left(\sin\theta \frac{\partial P}{\partial \theta }\right)+\frac{1}{\Phi }\frac{\partial^{2}\Phi }{\partial \varphi^{2}}=-\sin^{2}\theta \left[L\left(L+1\right)\right]$$

$$\frac{\sin\theta }{P}\frac{\partial }{\partial \theta }\left(\sin\theta \frac{\partial P}{\partial \theta }\right)+\sin^{2}\theta \left[L\left(L+1\right)\right]=-\frac{1}{\Phi }\frac{\partial^{2}\Phi }{\partial \varphi^{2}}=m^{2}$$

thus,

$$\frac{\sin\theta }{P}\frac{\partial }{\partial \theta }\left(\sin\theta \frac{\partial P}{\partial \theta }\right)+\sin^{2}\theta \left[L\left(L+1\right)\right]=m^{2}$$

Again, we multiply equation above by $\frac{P}{\sin^{2}\theta }$

(30) $$\frac{1}{\sin\theta }\frac{\partial }{\partial \theta }\left(\sin\theta \frac{\partial P}{\partial \theta }\right)+L(L+1)P-\frac{m^{2}}{\sin^{2}\theta }P=0$$

and

(31) $$\frac{\partial^{2}\Phi }{\partial \varphi^{2}}+m^{2}\Phi =0$$

The Woods Saxon potential [28], [29], [64] in general form appears as

(54) $$V\left(\beta \right)=-\frac{V_{0}}{1+e^{\frac{\beta -\beta_{0}}{r_{0}}}}$$

The potential in equation (30) could be simplified by setting

(55) $$e^{\frac{-\beta_{0}}{r_{0}}}=\frac{1}{\sigma }$$

Then, the Woods-Saxon potential turns to the following form

$$V\left(\beta \right)=-\frac{V_{0}}{1+\frac{1}{\sigma }e^{\frac{\beta }{r_{0}}}}$$

The latter equation is multiplied by $e^{-\frac{\beta }{2r_{0}}}$ gives

(56) $$V\left(\beta \right)=-\frac{\sigma V_{0}e^{-\frac{\beta }{2r_{0}}}}{\left(\sigma e^{-\frac{\beta }{2r_{0}}}+e^{\frac{\beta }{2r_{0}}}\right)}$$

By inserting equation (56) into equation (28), the radial part of Bohr Mottelson Hamiltonian equation within the minimal length formalism changes in the following condition

(74) $$\frac{\partial^{2}R}{\partial \beta^{2}}-\frac{\omega }{\mu }\frac{\left(\tau +\varepsilon \right)\left(-\frac{\sigma V_{0}e^{-\frac{\beta }{2r_{0}}}}{\left(\sigma e^{-\frac{\beta }{2r_{0}}}+e^{\frac{\beta }{2r_{0}}}\right)}\right)}{\left(1+\tau \left(-\frac{\sigma V_{0}e^{-\frac{\beta }{2r_{0}}}}{\left(\sigma e^{-\frac{\beta }{2r_{0}}}+e^{\frac{\beta }{2r_{0}}}\right)}\right)\right)}R+\frac{\omega }{\mu }R-\frac{\delta }{3\beta^{2}}R=0$$

Equation (74) can be rewritten as

(75) $$\frac{\partial^{2}R}{\partial \beta^{2}}-\frac{\omega }{\mu }\frac{\left(\tau +\varepsilon \right)\left(-\sigma V_{0}e^{-\frac{\beta }{2r_{0}}}\right)}{\left(e^{\frac{\beta }{2r_{0}}}-\sigma \left(\tau V_{0}-1\right)e^{-\frac{\beta }{2r_{0}}}\right)}R+\frac{\omega }{\mu }R-\frac{\delta }{3\beta^{2}}R=0$$

in equation (75), the new parameters should be set as

(76) $$\sigma \left(\tau V_{0}-1\right)=q;\frac{1}{2r_{0}}=\gamma^{\prime }$$

And by using equations (57), (60), and (76), equation (65) leads to

$$\frac{\partial^{2}R}{\partial \beta^{2}}+\frac{\omega }{\mu }\frac{\left(\tau +\varepsilon \right)\sigma V_{0}\left(\cosh\gamma^{\prime }\beta -\sinh\gamma^{\prime }\beta \right)}{\left(2q\right)\left(\sinh\gamma^{\prime }\beta \right)}R+\frac{\omega }{\mu }R-\frac{\delta \gamma^{\prime \;2}}{3\sinh^{2}\gamma^{\prime }\beta }R=0$$

(77) $$\frac{\partial^{2}R}{\partial \beta^{2}}+\frac{\omega }{\mu }\frac{\left(\tau +\varepsilon \right)\sigma V_{0}}{\left(2q\right)}\left(\coth\gamma^{\prime }\beta -1\right)R+\frac{\omega }{\mu }R-\frac{\delta \gamma^{\prime \;2}}{3\sinh^{2}\gamma^{\prime }\beta }R=0$$

The equation yields

(78) $$\frac{\partial^{2}R}{\partial \beta^{2}}-\left(\frac{\gamma^{\prime \;2}L^{\prime }\left(L^{\prime }-1\right)}{\sinh^{2}\gamma^{\prime }\beta }-2\eta \left(\coth\gamma^{\prime }\beta \right)-E^{\prime }\right)R=0$$

with

(79) $$\frac{\delta }{3}=\frac{L(L+1)}{3}=L^{\prime }(L^{\prime }-1);\;\frac{\omega }{\mu }\frac{\left(\tau +\varepsilon \right)\sigma V_{0}}{\left(2q\right)}=2\eta ;\;-\frac{\omega }{\mu }\frac{\left(\tau +\varepsilon \right)\sigma V_{0}}{\left(2q\right)}+\frac{\omega }{\mu }=E^{\prime }$$

and we use the approximate value of $\frac{1}{\beta^{2}}$ [24] given as

(80) $$\frac{1}{\beta^{2}}=\frac{\gamma^{\prime \;2}}{\sinh^{2}\gamma^{\prime }\beta }$$

To solve equation (78), we should set a new variable that is

(81) $$\coth\gamma^{\prime }\beta =1-2z$$

$$\frac{d}{d\beta }=\frac{d}{dz}\frac{dz}{d\beta }=2\gamma^{\prime }\left(z^{2}-z\right)\frac{d}{dz}$$

(82) $$\frac{d^{2}}{d\beta^{2}}=4\gamma^{\prime \;2}z^{2}{\left(z-1\right)}^{2}\frac{d^{2}}{dz^{2}}+4\gamma^{\prime \;2}z\left(z-1\right)\left(2z-1\right)\frac{d}{dz}$$

Equation (81) and (82) are inserted into equation (78), we obtain

(83) $$4\gamma^{\prime \;2}z^{2}{\left(z-1\right)}^{2}\frac{d^{2}R\left(\beta \right)}{dz^{2}}+4\gamma^{\prime \;2}z\left(z-1\right)\left(2z-1\right)\frac{dR\left(\beta \right)}{dz}-\left[\begin{array}{c} 4\gamma^{\prime \;2}L^{\prime }\left(L^{\prime }-1\right)z\left(z-1\right)-\\ 2\eta \left(1-2z\right)-E^{\prime } \end{array}\right]R\left(\beta \right)=0$$

For mathematical simplicity, we renaming the parameters according to

(84) $$\frac{\eta }{\gamma^{\prime \;2}}=\eta^{\prime };\;-\frac{E^{\prime }}{\gamma^{\prime \;2}}=k^{2}$$

If we divide each term in equation (83) by $4\gamma^{\prime \;2}\;z\left(z-1\right)$ we obtain

$$z\left(1-z\right)\frac{d^{2}R}{dz^{2}}+\left(1-2z\right)\frac{dR}{dz}+\left[L^{\prime }\left(L^{\prime }-1\right)-\frac{2\eta^{\prime }\left(1-2z\right)}{4z\left(1-z\right)}-\frac{k^{2}}{4z\left(1-z\right)}\right]R=0$$

(85) $$z\left(1-z\right)\frac{d^{2}R}{dz^{2}}+\left(1-2z\right)\frac{dR}{dz}+\left[L^{\prime }\left(L^{\prime }-1\right)-\frac{-2\eta^{\prime }+k^{2}}{4z}-\frac{2\eta^{\prime }+k^{2}}{4\left(1-z\right)}\right]R=0$$

To apply the hypergeometric method, we propose a new wave function as follows

(86) $$R\left(\beta \right)=z^{\alpha }{\left(1-z\right)}^{\vartheta }f\left(z\right)$$

And we use the following new parameters

(87) $$-2\eta^{\prime }+k^{2}=4\alpha^{2};2\eta^{\prime }+k^{2}=4\vartheta^{2}$$

Solving equation (85) by deriving of $\frac{dR\left(\beta \right)}{dz}$

$$\frac{d\left[z^{\alpha }{\left(1-z\right)}^{\vartheta }f\left(z\right)\right]}{dz}=\alpha z^{\alpha -1}{\left(1-z\right)}^{\vartheta }f\left(z\right)-z^{\alpha }\vartheta {\left(1-z\right)}^{\vartheta -1}f\left(z\right)+z^{\alpha }{\left(1-z\right)}^{\vartheta }f^{\prime }\left(z\right)$$

While the derivation of $\frac{d^{2}R\left(\beta \right)}{dz^{2}}$ is

$$\frac{d\left[\alpha z^{\alpha -1}{\left(1-z\right)}^{\vartheta }f\left(z\right)-z^{\alpha }\vartheta {\left(1-z\right)}^{\vartheta -1}f\left(z\right)+z^{\alpha }{\left(1-z\right)}^{\vartheta }f^{\prime }\left(z\right)\right]}{dz}=\frac{d\left[A+B+C\right]}{dz}$$

$$\frac{dA}{dz}=\frac{d\left[\alpha z^{\alpha -1}{\left(1-z\right)}^{\vartheta }f\left(z\right)\right]}{dz}=\alpha \left(\alpha -1\right)z^{\alpha -2}{\left(1-z\right)}^{\vartheta }f\left(z\right)-\alpha z^{\alpha -1}\vartheta {\left(1-z\right)}^{\vartheta -1}f\left(z\right)+\alpha z^{\alpha -1}{\left(1-z\right)}^{\vartheta }f^{\prime }\left(z\right)$$

$$\frac{dB}{dz}=\frac{d\left[-z^{\alpha }\vartheta {\left(1-z\right)}^{\vartheta -1}f\left(z\right)\right]}{dz}=-\alpha z^{\alpha -1}\vartheta {\left(1-z\right)}^{\vartheta -1}f\left(z\right)+z^{\alpha }\vartheta \left(\vartheta -1\right){\left(1-z\right)}^{\vartheta -2}f\left(z\right)-z^{\alpha }\vartheta {\left(1-z\right)}^{\vartheta -1}f^{\prime }\left(z\right)\frac{dC}{dz}=\frac{d\left[z^{\alpha }{\left(1-z\right)}^{\vartheta }f^{\prime }\left(z\right)\right]}{dz}=\alpha z^{\alpha -1}{\left(1-z\right)}^{\vartheta }f^{\prime }\left(z\right)-z^{\alpha }\vartheta {\left(1-z\right)}^{\vartheta -1}f^{\prime }\left(z\right)+z^{\alpha }{\left(1-z\right)}^{\vartheta }f^{\prime \prime }\left(z\right)$$

Thus,

$$\frac{d^{2}R\left(\beta \right)}{dz^{2}}=\frac{dA}{dz}+\frac{dB}{dz}+\frac{dC}{dz}=\left[\begin{array}{c} \alpha \left(\alpha -1\right)z^{\alpha -2}{\left(1-z\right)}^{\vartheta }f\left(z\right)-\alpha z^{\alpha -1}\vartheta {\left(1-z\right)}^{\vartheta -1}f\left(z\right)+\alpha z^{\alpha -1}{\left(1-z\right)}^{\vartheta }f^{\prime }\left(z\right)\\ -\alpha z^{\alpha -1}\vartheta {\left(1-z\right)}^{\vartheta -1}f\left(z\right)+z^{\alpha }\vartheta \left(\vartheta -1\right){\left(1-z\right)}^{\vartheta -2}f\left(z\right)-z^{\alpha }\vartheta {\left(1-z\right)}^{\vartheta -1}f^{\prime }\left(z\right)\\ +\alpha z^{\alpha -1}{\left(1-z\right)}^{\vartheta }f^{\prime }\left(z\right)-z^{\alpha }\vartheta {\left(1-z\right)}^{\vartheta -1}f^{\prime }\left(z\right)+z^{\alpha }{\left(1-z\right)}^{\vartheta }f^{\prime \prime }\left(z\right) \end{array}\right]$$

$$\frac{d^{2}R\left(\beta \right)}{dz^{2}}=\left[\begin{array}{c} \alpha \left(\alpha -1\right)z^{\alpha -2}{\left(1-z\right)}^{\vartheta }f\left(z\right)-2\alpha z^{\alpha -1}\vartheta {\left(1-z\right)}^{\vartheta -1}f\left(z\right)+2\alpha z^{\alpha -1}{\left(1-z\right)}^{\vartheta }f^{\prime }\left(z\right)\\ +z^{\alpha }\vartheta \left(\vartheta -1\right){\left(1-z\right)}^{\vartheta -2}f\left(z\right)-2z^{\alpha }\vartheta {\left(1-z\right)}^{\vartheta -1}f^{\prime }\left(z\right)+z^{\alpha }{\left(1-z\right)}^{\vartheta }f^{\prime \prime }\left(z\right) \end{array}\right]$$

$$\frac{d^{2}R\left(\beta \right)}{dz^{2}}=\left[\begin{array}{c} \frac{\alpha \left(\alpha -1\right)}{z^{2}}z^{\alpha }{\left(1-z\right)}^{\vartheta }f\left(z\right)-\frac{2\alpha \vartheta }{z\left(1-z\right)}z^{\alpha }{\left(1-z\right)}^{\vartheta }f\left(z\right)+\frac{2\alpha }{z}z^{\alpha }{\left(1-z\right)}^{\vartheta }f^{\prime }\left(z\right)\\ +\frac{\vartheta \left(\vartheta -1\right)}{{\left(1-z\right)}^{2}}z^{\alpha }{\left(1-z\right)}^{\vartheta }f\left(z\right)-\frac{2\vartheta }{\left(1-z\right)}z^{\alpha }\vartheta {\left(1-z\right)}^{\vartheta }f^{\prime }\left(z\right)+z^{\alpha }{\left(1-z\right)}^{\vartheta }f^{\prime \prime }\left(z\right) \end{array}\right]$$

Equation (61) becomes

$$z\left(1-z\right)\left[\begin{array}{c} \frac{\alpha \left(\alpha -1\right)}{z^{2}}z^{\alpha }{\left(1-z\right)}^{\vartheta }f\left(z\right)-\frac{2\alpha \vartheta }{z\left(1-z\right)}z^{\alpha }{\left(1-z\right)}^{\vartheta }f\left(z\right)+\frac{2\alpha }{z}z^{\alpha }{\left(1-z\right)}^{\vartheta }f^{\prime }\left(z\right)\\ +\frac{\vartheta \left(\vartheta -1\right)}{{\left(1-z\right)}^{2}}z^{\alpha }{\left(1-z\right)}^{\vartheta }f\left(z\right)-\frac{2\vartheta }{\left(1-z\right)}z^{\alpha }\vartheta {\left(1-z\right)}^{\vartheta }f^{\prime }\left(z\right)+z^{\alpha }{\left(1-z\right)}^{\vartheta }f^{\prime \prime }\left(z\right) \end{array}\right]+\left(1-2z\right)\left[\begin{array}{c} \frac{\alpha }{z}z^{\alpha }{\left(1-z\right)}^{\vartheta }f\left(z\right)\\ -\frac{\vartheta }{\left(1-z\right)}z^{\alpha }{\left(1-z\right)}^{\vartheta }f\left(z\right)\\ +z^{\alpha }{\left(1-z\right)}^{\vartheta }f^{\prime }\left(z\right) \end{array}\right]+\left[L^{\prime }\left(L^{\prime }-1\right)-\frac{4\alpha^{2}}{4z}-\frac{4\vartheta^{2}}{4\left(1-z\right)}\right]\left[z^{\alpha }{\left(1-z\right)}^{\vartheta }f\left(z\right)\right]=0$$

It is divided by $z^{\alpha }{\left(1-z\right)}^{\vartheta }$

$$z\left(1-z\right)\left[\begin{array}{c} \frac{\alpha \left(\alpha -1\right)}{z^{2}}f\left(z\right)-\frac{2\alpha \vartheta }{z\left(1-z\right)}f\left(z\right)+\frac{2\alpha }{z}f^{\prime }\left(z\right)\\ +\frac{\vartheta \left(\vartheta -1\right)}{{\left(1-z\right)}^{2}}f\left(z\right)-\frac{2\vartheta }{\left(1-z\right)}f^{\prime }\left(z\right)+f^{\prime \prime }\left(z\right) \end{array}\right]+\left(1-2z\right)\left[\frac{\alpha }{z}f\left(z\right)-\frac{\vartheta }{\left(1-z\right)}f\left(z\right)+f^{\prime }\left(z\right)\right]+\left[L^{\prime }\left(L^{\prime }-1\right)-\frac{4\alpha^{2}}{4z}-\frac{4\vartheta^{2}}{4\left(1-z\right)}\right]f\left(z\right)=0$$

It can be rewritten as

$$\left[\begin{array}{c} \frac{\alpha \left(\alpha -1\right)}{z}f\left(z\right)-\alpha \left(\alpha -1\right)f\left(z\right)-2\alpha \vartheta f\left(z\right)\\ +2\alpha \left(1-z\right)f^{\prime }\left(z\right)+\frac{\vartheta \left(\vartheta -1\right)z}{\left(1-z\right)}f\left(z\right)-2\vartheta zf^{\prime }\left(z\right)+z\left(1-z\right)f^{\prime \prime }\left(z\right) \end{array}\right]+\left[\begin{array}{c} \frac{\alpha }{z}f\left(z\right)-2\alpha f\left(z\right)-\frac{\vartheta \left(1-2z\right)}{\left(1-z\right)}f\left(z\right)\\ +\left(1-2z\right)f^{\prime }\left(z\right) \end{array}\right]+\left[L^{\prime }\left(L^{\prime }-1\right)-\frac{4\alpha^{2}}{4z}-\frac{4\vartheta^{2}}{4\left(1-z\right)}\right]f\left(z\right)=0$$

where,

$$\frac{\vartheta \left(\vartheta -1\right)z}{\left(1-z\right)}=\frac{\vartheta \left(\vartheta -1\right)}{\left(1-z\right)}-\frac{\left(1-z\right)\vartheta \left(\vartheta -1\right)}{\left(1-z\right)}=\frac{\vartheta \left(\vartheta -1\right)}{\left(1-z\right)}-\vartheta \left(\vartheta -1\right)$$

and

$$\frac{\vartheta \left(1-2z\right)}{\left(1-z\right)}=\frac{2\vartheta \left(1-z\right)}{\left(1-z\right)}-\frac{\vartheta }{\left(1-z\right)}=2\vartheta -\frac{\vartheta }{\left(1-z\right)}$$

So, we have the form

$$\left[\begin{array}{c} \frac{\alpha \left(\alpha -1\right)}{z}-\alpha \left(\alpha -1\right)-2\alpha \vartheta +\frac{\vartheta \left(\vartheta -1\right)}{\left(1-z\right)}\\ -\vartheta \left(\vartheta -1\right)+\frac{\alpha }{z}-2\alpha -2\vartheta \\ +\frac{\vartheta }{\left(1-z\right)}+L^{\prime }\left(L^{\prime }-1\right)-\frac{4\alpha^{2}}{4z}-\frac{4\vartheta^{2}}{4\left(1-z\right)} \end{array}\right]f\left(z\right)+\left[2\alpha \left(1-z\right)-2\vartheta z+\left(1-2z\right)\right]f^{\prime }\left(z\right)+z\left(1-z\right)f^{\prime \prime }\left(z\right)=0$$

where,

$$\frac{\alpha \left(\alpha -1\right)}{z}+\frac{\alpha }{z}-\frac{4\alpha^{2}}{4z}=\frac{4\alpha^{2}-4\alpha +4\alpha -4\alpha^{2}}{4z}=0$$

and

$$\frac{\vartheta \left(\vartheta -1\right)}{\left(1-z\right)}+\frac{\vartheta }{\left(1-z\right)}-\frac{4\vartheta^{2}}{4\left(1-z\right)}=\frac{4\vartheta^{2}-4\vartheta +4\vartheta -4\vartheta^{2}}{\left(1-z\right)}=0$$

From the latter equation, we obtain the hypergeometric differential equation given as

(88) $$z\left(1-z\right)f^{\prime \prime }\left(z\right)+\left[\left(2\alpha +1\right)-\left(2\alpha +2\vartheta +2\right)z\right]f^{\prime }\left(z\right)-\left[\begin{array}{c} \left(\alpha +\vartheta \right)\left(\alpha +\vartheta +1\right)\\ -L^{\prime }\left(L^{\prime }-1\right) \end{array}\right]f\left(z\right)=0$$

By comparing equations (70) and (88), we obtain

(89) $$a^{\prime }=\alpha +\vartheta -L^{\prime }+1;\;b^{\prime }=\alpha +\vartheta +L^{\prime };c^{\prime }=2\alpha +1$$

Since we take $a^{\prime }=-n$, then equation (89) gives

(90) $$\alpha +\vartheta =L^{\prime }-1-n\to \alpha^{2}+2\alpha \vartheta +\vartheta^{2}={\left(L^{\prime }-1-n\right)}^{2}$$

Therefore, the energy spectra are obtained from equation (87), (90), and (88) as the form

$$\alpha^{2}+2\alpha \vartheta +\vartheta^{2}={\left(L^{\prime }-1-n\right)}^{2}\;\frac{-2\eta^{\prime }+k^{2}}{4}+2\sqrt{\frac{-2\eta^{\prime }+k^{2}}{4}}\sqrt{\frac{2\eta^{\prime }+k^{2}}{4}}+\frac{2\eta^{\prime }+k^{2}}{4}={\left(L^{\prime }-1-n\right)}^{2};\frac{2k^{2}}{4}+2\sqrt{\frac{1}{16}\left(k^{2}-4\eta^{\prime \;2}\right)}={\left(L^{\prime }-1-n\right)}^{2}\;\frac{1}{2}\sqrt{\left(k^{2}-4\eta^{\prime \;2}\right)}={\left(L^{\prime }-1-n\right)}^{2}-\frac{k^{2}}{2}{\left[\frac{1}{2}\sqrt{\left(k^{2}-4\eta^{\prime \;2}\right)}\right]}^{2}={\left[{\left(L^{\prime }-1-n\right)}^{2}-\frac{k^{2}}{2}\right]}^{2}\;\frac{1}{4}\left(k^{2}-4\eta^{\prime \;2}\right)={\left(L^{\prime }-1-n\right)}^{4}-k^{2}{\left(L^{\prime }-1-n\right)}^{2}+\frac{k^{4}}{4}\;k^{2}{\left(L^{\prime }-1-n\right)}^{2}={\left(L^{\prime }-1-n\right)}^{4}+\eta^{\prime \;2}$$

(91) $$k^{2}={\left(L^{\prime }-1-n\right)}^{2}+\frac{\eta^{\prime \;2}}{{\left(L^{\prime }-1-n\right)}^{2}}$$

Using equations (79), (84), and (91), the energy spectra equation yields

(92) $$\frac{\omega }{\gamma^{\prime \;2}\mu }\left(\frac{\left(\tau +\varepsilon \right)\sigma V}{2q}-1\right)={\left(L^{\prime }-1-n\right)}^{2}+\left[\frac{\frac{\omega }{\gamma^{\prime \;2}\mu }{\left(\frac{\left(\tau +\varepsilon \right)\sigma V}{2q}\right)}^{2}}{{\left(L^{\prime }-1-n\right)}^{2}}\right]=0$$

Rewriting equation (92) by inserting equation (22), we have

(93) $$\frac{\frac{2B_{m}}{\hbar^{2}}E}{\gamma^{\prime \;2}\left(1+4B_{m}\alpha_{\mathrm{ML}}E\right)}\left[\frac{\left(\frac{1}{E}-\frac{4B_{m}\alpha_{\mathrm{ML}}}{1+4B_{m}\alpha_{\mathrm{ML}}E}\right)\sigma V_{0}}{2q}-1\right]=\left[\begin{array}{c} {\left(L^{\prime }-1-n\right)}^{2}+\\ \frac{{\left(\frac{\frac{2B_{m}}{\hbar^{2}}E}{\gamma^{\prime \;2}\left(1+4B_{m}\alpha_{\mathrm{ML}}E\right)}\frac{\left(\frac{1}{E}-\frac{4B_{m}\alpha_{\mathrm{ML}}}{1+4B_{m}\alpha_{\mathrm{ML}}E}\right)\sigma V_{0}}{4q}\right)}^{2}}{{\left(L^{\prime }-1-n\right)}^{2}} \end{array}\right]$$

The above formulation leads us to the energy equation as

(94) $$E_{n}=-\frac{4\gamma^{\prime \;2}\left(1+4B_{m}\alpha_{\mathrm{ML}}E\right)}{\frac{2B_{m}}{\hbar^{2}}}\left[\begin{array}{c} \frac{{\left(n+1-L^{\prime }\right)}^{2}}{4}+\frac{{\left(\frac{{\left(\frac{2B_{m}}{\hbar^{2}}\sigma V_{0}\right)}^{2}}{\gamma^{\prime \;4}\left(4\right)\left(16q^{2}\right){\left(1+4B_{m}\alpha_{\mathrm{ML}}E\right)}^{4}}\right)}^{2}}{{\left(n+1-L^{\prime }\right)}^{2}}-\\ \frac{2B_{m}\sigma V_{0}}{2\left(4q\right){\left(1+4B_{m}\alpha_{\mathrm{ML}}E\right)}^{2}} \end{array}\right]$$

that gives

(95) $$E_{n}=-\frac{4\gamma^{\prime \;2}\left(1+4B_{m}\alpha_{\mathrm{ML}}E\right)}{\frac{2B_{m}}{\hbar^{2}}}{\left[\frac{\left(n+1-L^{\prime }\right)}{2}+\frac{\left(\frac{{\left(\frac{B_{m}}{\hbar^{2}}\sigma V_{0}\right)}^{2}}{4\gamma^{\prime \;4}q{\left(1+4B_{m}\alpha_{\mathrm{ML}}E\right)}^{2}}\right)}{\left(n+1-L^{\prime }\right)}\right]}^{2}$$

With $L^{\prime }$ is obtained using equation (79)

$$\frac{L(L+1)}{3}=L^{\prime }(L^{\prime }-1)\frac{L(L+1)}{3}+\frac{1}{4}=L^{\prime }(L^{\prime }-1)+\frac{1}{4}\;\sqrt{\frac{L(L+1)}{3}+\frac{1}{4}}={\left(L^{\prime }-\frac{1}{2}\right)}^{2}\;L^{\prime }=\frac{1}{2}+\sqrt{\frac{L\left(L+1\right)}{3}+\frac{1}{4}}$$
